# The Transposition Rate Has Little Influence on the Plateauing Level of the P-element

**DOI:** 10.1093/molbev/msac141

**Published:** 2022-06-22

**Authors:** Robert Kofler, Viola Nolte, Christian Schlötterer

**Affiliations:** Institut für Populationsgenetik, Vetmeduni Vienna, Veterinärplatz 1, 1210 Wien, Austria; Institut für Populationsgenetik, Vetmeduni Vienna, Veterinärplatz 1, 1210 Wien, Austria; Institut für Populationsgenetik, Vetmeduni Vienna, Veterinärplatz 1, 1210 Wien, Austria

**Keywords:** transposable elements, P-element, population genetics, experimental evolution, Drosophila simulans

## Abstract

The popular trap model assumes that the invasions of transposable elements (TEs) in mammals and invertebrates are stopped by piRNAs that emerge after insertion of the TE into a piRNA cluster. It remains, however, still unclear which factors influence the dynamics of TE invasions. The activity of the TE (i.e., transposition rate) is one frequently discussed key factor. Here we take advantage of the temperature-dependent activity of the P-element, a widely studied eukaryotic TE, to test how TE activity affects the dynamics of a TE invasion. We monitored P-element invasion dynamics in experimental *Drosophila simulans* populations at hot and cold culture conditions. Despite marked differences in transposition rates, the P-element reached very similar copy numbers at both temperatures. The reduction of the insertion rate upon approaching the copy number plateau was accompanied by similar amounts of piRNAs against the P-element at both temperatures. Nevertheless, we also observed fewer P-element insertions in piRNA clusters than expected, which is not compatible with a simple trap model. The ping-pong cycle, which degrades TE transcripts, becomes typically active after the copy number plateaued. We generated a model, with few parameters, that largely captures the observed invasion dynamics. We conclude that the transposition rate has at the most only a minor influence on TE abundance, but other factors, such as paramutations or selection against TE insertions are shaping the TE composition.

## Introduction

Transposable elements (TEs) are short stretches of DNA that spread within genomes, even at the cost of host fitness (Doolittle and Sapienza 1980; Orgel and Crick 1980; Hickey 1982). TEs are found in most prokaryotic and eukaryotic genomes (Biémont and Vieira 2006; Wicker et al. 2007), and they frequently invade new species by horizontal transfer (Loreto et al. 2008; El Baidouri and Panaud 2013; Peccoud et al. 2017). After horizontal transfer a TE may spread in a naive species until all populations have acquired the TE. The increase in copy number of the TEs is counteracted by small RNAs and negative selection (Nuzhdin 1999; Barrón et al. 2014; Brennecke et al. 2007). TEs may be deleterious to hosts and thus negatively selected due to three mechanisms: (1) TE insertions could directly disrupt genes or promoter regions, (2) ectopic recombination between insertions at different sites could lead to deleterious genomic rearrangements, and (3) the products of TEs such as the transposase could be deleterious [e.g., by generating DNA damage as found during hybrid dysgenesis (Nuzhdin 1999; Moon et al. 2018)].

In mammals and invertebrates, the defence against TEs is mediated by piRNAs, small RNAs ranging in size between 23 and 29 nt (Brennecke et al. 2007; Gunawardane et al. 2007; Czech and Hannon 2016; Ozata et al. 2018). These piRNAs interact with PIWI clade proteins and silence the TEs at the transcriptional and the post-transcriptional level (Brennecke et al. 2007; Gunawardane et al. 2007; Sienski et al. 2012; Le Thomas et al. 2013). piRNAs are largely derived from discrete genomic loci called piRNA clusters (Brennecke et al. 2007; Gebert et al. 2021). piRNA clusters are usually found in the heterochromatin, close to the euchromatin boundary, and may constitute substantial portions of genomes (Brennecke et al. 2007). In *D. melanogaster*, for example, piRNA clusters account for about 3.5% of the genome (Brennecke et al. 2007). The trap model proposes that an invading TE multiplies until one copy jumps into a piRNA cluster, which triggers the production of piRNAs that silence invading TEs (Bergman et al. 2006; Malone and Hannon 2009; Zanni et al. 2013; Goriaux et al. 2014; Yamanaka et al. 2014; Ozata et al. 2018; Duc et al. 2019).

Apart from negative selection and small RNAs, which counteract TEs, other factors may impact the dynamics of TE invasion (i.e., the spread of a TE in a population). One frequently discussed factor is the transposition rate, that is, the probability that a TE insertion generates a novel copy in the next generation (Charlesworth and Charlesworth 1983; Nuzhdin 1999; Kofler 2019; Kelleher et al. 2020). The transposition rate varies substantially among TE families (Nuzhdin 1999; Robillard et al. 2016; Kofler et al. 2018), and it was long considered to be the major factor determining the abundance of TEs in organisms (Charlesworth and Charlesworth 1983; Kofler, Nolte, et al. 2015). The impact of the transposition rate depends on the mechanism by which TE invasions are controlled. It was previously assumed that TE invasions are mostly counteracted by negative selection (Charlesworth and Charlesworth 1983; Charlesworth and Langley 1989). If negative selection against TEs increases exponentially with TE copy numbers an equilibrium may be reached where the same number of TE insertions are acquired by transposition as are lost by negative selection (Charlesworth and Charlesworth 1983; Charlesworth and Langley 1989; Barrón et al. 2014) [other conditions for an equilibrium are also feasible (Charlesworth and Charlesworth 1983)]. The TE abundance at this equilibrium depends on the transposition rate (Kofler, Nolte, et al. 2015). For the trap model, a non-equilibrium model, that assumes that an active TE is silenced by a TE insertion in a piRNA cluster, the transposition rate may only have a minor influence on the level at which TE copy numbers stabilize (i.e., the plateau level) (Kelleher et al. 2018; Kofler 2019).

Although these two models generate clear predictions about the role of the transposition rate, several lines of evidence suggest a more complex scenario. First, a lag-time (e.g., 7 generations) between the emergence of a cluster insertion and the establishment of an effective piRNA-based defence (Josse et al. 2007) could cause an effect of transposition rate, even without selection. Second, if both negative selection and cluster insertions control TE invasions, complex 3-way interactions among negative selection, transposition and piRNA clusters may arise where the transposition rate influences the abundance of TEs at the equilibrium level (Kofler 2019). Finally, it is conceivable that the trap model does not hold. In *D. melanogaster* cluster insertions may not be required for maintaining silencing of TEs (Gebert et al. 2021) and in koalas the initial piRNAs against an invading TE arise even without cluster insertion (Yu et al. 2019). The role of the transposition rate in alternative models of TE silencing are yet unclear. Despite this potentially important role of the transposition rate, empirical evidence has been difficult to obtain. Comparing invasions of different TE families with varying transposition rates will lead to inconclusive results, as the families also differ in many other factors that influence invasion dynamics such as insertion biases (e.g., into repetitive regions, genes, promoters), mutation rate (including internal deletions) and the fitness consequences to the host (Gdula et al. 1996; Spradling et al. 2011; Burt and Trivers 2008; Feschotte 2008; Sultana et al. 2017).

Here we propose an elegant solution to overcome these limitations. As the activity of many TEs depends on the temperature (Jakšić et al. 2017; Kofler et al. 2018; Moon et al. 2018) we may study the influence of the transposition rate on invasion dynamics while minimizing the impact of other factors by modulating the ambient temperature during a TE invasion. Especially the P-element, one of the best-studied eukaryotic TEs (Engels 1983; Kelleher 2016), is ideally suited for this task. It is a DNA transposon (cut-and-paste) with a length of 2907 bp encoding a single protein, the transposase (Bingham et al. 1982; O’Hare and Rubin 1983; Finnegan 1989). The P-element increases its copy number by sister chromatid-mediated gap repair after the excision of a P-element (Engels et al. 1990) combined with preferential insertion into unreplicated DNA (Spradling et al. 2011). Interestingly, due to tissue-specific alternative splicing of its third intron (IVS3) the P-element is solely active in the germline (Laski et al. 1986), which is thought to minimize damage of the P-element to the host (Burt and Trivers 2008). The P-element is highly invasive, having invaded natural populations of *D. melanogaster* as well as *D. simulans* in the last 100 years (Daniels et al. 1990; Kofler, Hill, et al. 2015; Hill et al. 2016). The P-element is also responsible for the well-described hybrid dysgenesis syndrome (HD), where crosses of males having the P-element with females not-having it lead to offspring with atrophied ovaries (and other symptoms), whereas reciprocal crosses produce fertile offspring (Kidwell et al. 1977; Bingham et al. 1982; Kelleher 2016). It was also discovered that atrophied ovaries are solely observed in crosses performed at high temperatures (Kidwell et al. 1977; Kidwell and Novy 1979; Moon et al. 2018). This can be explained by atrophied ovaries as a consequence of P-element activity (Moon et al. 2018), which increases with temperature (Kaufman and Rio 1992; Kofler et al. 2018; Moon et al. 2018).

To investigate the impact of the transposition rate on invasion dynamics, we studied P-element invasion at two different temperatures (hot and cold conditions) in experimental *D. simulans* populations. Our analyses confirm substantially different transposition rates at the two temperatures, but the abundance of the P-element plateaus at almost the same level. This plateauing of the P-element was accompanied by a rapid emergence of piRNAs against the P-element at both temperatures. All parameters characterizing the P-element invasion, such as insertion biases, the extent of genetic drift during the experiments, the proportion of insertions in functionally different genomic regions, the size of piRNA clusters, the fraction of insertions with internal deletions that could down-regulate the P-element, were quite similar between the two temperatures. Modeling the invasions in our populations we show that a simple model with piRNA clusters and negative selection against TEs largely captures the observed invasion dynamics.

## Results

### Invasion Dynamics of the P-Element

To investigate the role of the transposition rate (prior to the emergence of piRNAs) during TE invasions, we monitored the spread of the P-element in experimentally evolving *D. simulans* populations at hot (18–28°C) and cold conditions (10–20°C). Since the P-element activity depends on temperature (Kaufman and Rio 1992; Kofler et al. 2018; Moon et al. 2018) this setup allows us to investigate the influence of the transposition rate on invasion dynamics while minimizing the influence of other TE family-specific factors such as differences in transcription factor binding sites or insertion preferences. The experimental populations were established from 202 isofemale lines collected 2010 in Florida. The population from Florida was at an early stage of the P-element invasion where about 25–44% of the isofemale lines carried P-element insertions (Kofler, Hill, et al. 2015; Hill et al. 2016). We used three replicates, non-overlapping generations and a census population size between 1,000 and 1,250. Our previous analysis of this P-element invasion only covered 40 generations of the cold conditions, which was not sufficient to observe the plateauing of P-element copy numbers (Kofler et al. 2018). Here, we significantly extended the experiment by additional 60 generations, which corresponds to a total of 10 years of experimental evolution. We monitored several key parameters of the cold invasion including the degree of gonadal dysgenesis, the abundance of P-element insertions and the amount of piRNAs against the P-element. All data, previously published ones and newly generated ones, were (re)analyzed using the same pipeline to ensure comparability of the data.

**Fig. 1.**
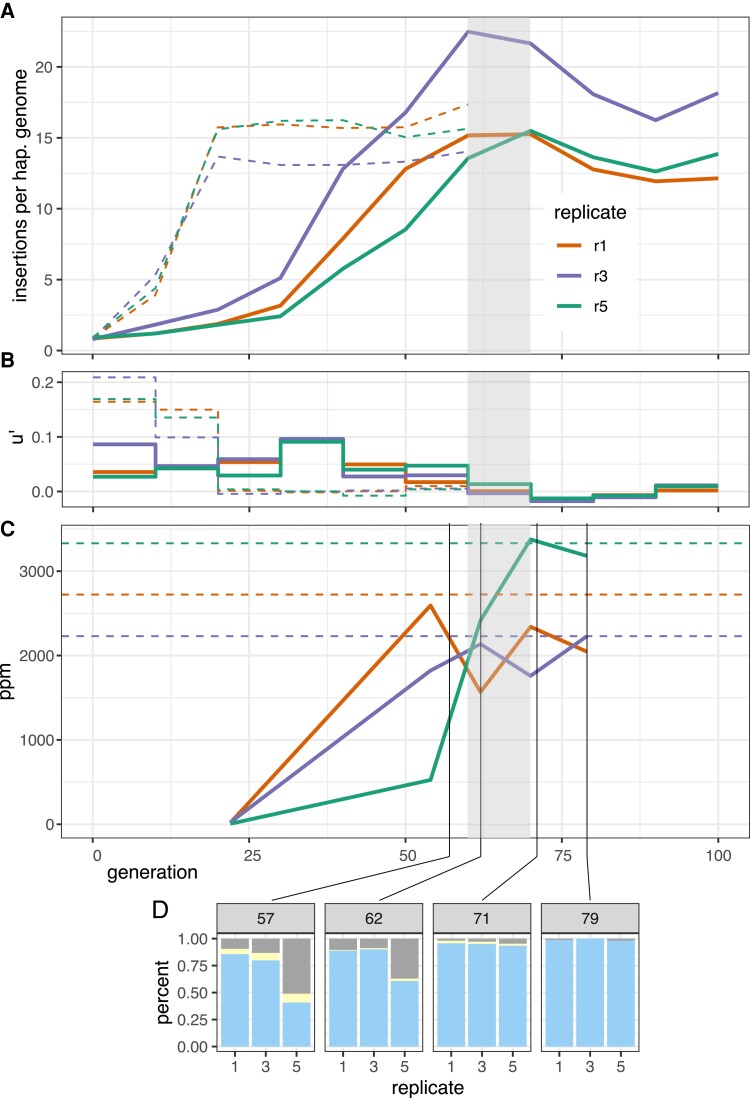
Dynamics of a natural P-element invasion in *D. simulans* at cold conditions (solid lines). (*A*) Abundance of P-element insertions during the invasion in three replicates. Hatched line shows the previously published invasion dynamics at hot conditions (Kofler et al. 2018). Grey shades indicate the proposed onset of the plateauing at cold conditions. (*B*) Effective transposition rates ($u^{\prime }$) during the invasions. (*C*) Abundance of piRNAs complementary to the P-element (ppm piRNAs per million miRNAs). Hatched lines show the average abundance of P-element piRNAs at the plateau (≥20 generations) of the hot invasion. Note that similar levels of piRNAs are necessary to stop the invasions at hot and cold conditions. (*D*) Abundance of dysgenic ovaries at 29°C. Blue, normal ovaries; grey, dysgenic ovaries; yellow, intermediate.

We first asked whether the P-element reached stable copy numbers in the cold invasion by generation 100. The experimental populations were sequenced in 10 generation intervals as pools (Pool-Seq (Schlötterer et al. 2014); for an overview of all genomic data used in this work see supplementary table S1, Supplementary Material online). The number of P-element insertions per haploid genome was estimated with DeviaTE, which normalizes the coverage of the P-element to the coverage of single-copy genes (Weilguny and Kofler 2019). The cold invasion reached a plateau of 12–22 P-element insertions per haploid genome around generations 60–70 (fig. 1*A* grey shades; supplementary table S2 and figs. S1–S3, Supplementary Material online). By contrast, the hot invasion reached stable copy numbers already at generation 20 with about 13–17 copies per haploid genome (fig. 1*A*; supplementary table S2 and figs. S4–S6, Supplementary Material online; data from Kofler et al. 2018). The copy numbers at the plateau were very similar between hot and cold conditions (Welch Two Sample *t*-test, hot generations 60 vs. cold generations 100, p=0.67).

We measured the effective transposition rates ($u^{\prime }=u-x$) during the invasion since we are not able to determine transposition rate (*u*) and selection (*x*) directly. The effective transposition rate differed between the hot and cold invasion (fig. 1*B*; supplementary table S2, Supplementary Material online). The hot invasion is characterized by a high transposition rate for the first 20 generations ($u^{\prime }\approx 0.099-0.209$) followed by a drop to about zero at later generations (fig. 1*B*; supplementary table S2, Supplementary Material online). In the cold invasion, the transposition rate is consistently lower than at hot conditions (0.017–0.096) and drops to values around zero after 60 generations (fig. 1*B*; supplementary table S2, Supplementary Material online). At the early stages of the invasions (without the influence of piRNAs), the effective transposition rates are significantly higher at hot than at cold conditions (fig. 1*B*; supplementary table S2, Supplementary Material online; Welch Two Sample *t*-test, generations 0–10, p=0.0058).

To obtain estimates of the TE abundance independent of DeviaTE, we also computed the normalized number of reads mapping to each TE (rpm; reads per million). Using this alternative approach, we obtained very similar invasion dynamics (supplementary fig. S7 and table S2, Supplementary Material online) and again found that the transposition rates are significantly higher at hot than at cold conditions (Welch Two Sample *t*-test, generations 0–10, p=0.0074), whereas the final abundance of the P-element is similar between the hot and the cold invasions (Welch Two Sample *t*-test: hot generation 60 vs. cold generation 100, p=0.96).

We thus conclude that the P-element reached similar copy numbers at the hot and the cold invasions despite transposition rates being significantly lower in cold than in hot conditions.

### piRNAs Against the P-Element Rapidly Emerged in Experimental Populations

According to the trap model the invasion of a TE is stopped when one copy of the TE inserts into a piRNA cluster, which triggers the production of piRNAs complementary to the TE. This prediction can be scrutinized by monitoring the emergence of piRNAs complementary to the P-element during the invasion. We analyzed small RNAs from whole bodies of female flies at generations 22, 54, 62, 70, 79 of the cold invasions and generations 22, 44, and 108 of the hot invasions (data from generations 62, 70, and 79 of the cold invasion were generated in this work; for an overview of all small RNA data used in this work see supplementary table S3, Supplementary Material online). In the cold invasions, the number of piRNAs complementary to the P-element rapidly increased between generations 22 and 62 (fig. 1*C*; supplementary table S4, Supplementary Material online), consistent with the decrease of the transposition rate after generation 60. Similarly to the hot invasion (Kofler et al. 2018) only the abundance and piRNA level of a single TE, that is, the P-element, increased during the cold invasion (supplementary fig. S12, Supplementary Material online). The piRNAs were mostly antisense to the P-element with a length between 23 and 29 nt (supplementary fig. S8, Supplementary Material online) and distributed over the entire P-element (supplementary fig. S9, Supplementary Material online).

**Fig. 2.**
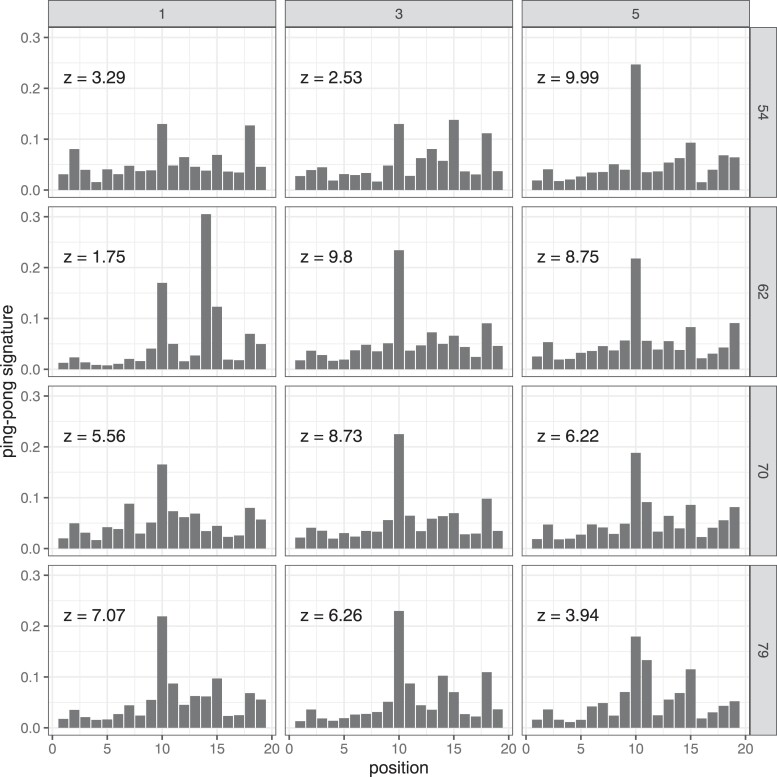
Ping-pong signature of P-element piRNAs during the cold invasion. We excluded piRNAs with $5^{\prime }$ positions 1162 and 1164 within the P-element. The *y*-axis shows the fraction of pairs of sense and antisense piRNAs having the given overlap (*x*-axis) between the two $5^{\prime }$ ends. Ping-pong *z*-scores are provided for each sample (e.g.: z=3.09 corresponds to p=0.001). Data are shown for the three replicates (top) at different generations during the experiment (right). Due to a low number of piRNAs we could not compute ping-pong signatures for any replicate at generation 22.

The piRNA response is amplified by the ping-pong cycle (Brennecke et al. 2007; Gunawardane et al. 2007), and we evaluated to what extent this process was driving the increase in piRNA copies during our experiment. The ping-pong cycle is based on two PIWI clade proteins Aub and Ago3. During the ping-pong cycle, RNA cleavage products of Aub are loaded onto Ago3 and vice versa. Since the Aub cleavage site is shifted by 10 bp from the Ago3 cleavage site a characteristic peak at position 10 can be found when plotting the average distance between the $5^{\prime }$ ends of sense and antisense piRNAs [i.e., the ping-pong signature; (Brennecke et al. 2007; Gunawardane et al. 2007)]. The size of the peak at position 10 (*h* the fraction of pairs of sense and antisenese piRNAs with the given distance) indicates the contribution of the ping-pong pathway to the total piRNA population. We found a peak at position 10 and an unexpected peak at position 12 (supplementary fig. S10, Supplementary Material online). We think that this peak at position 12 is an artifact of the uneven distribution of piRNAs along the P-element, since only a ping-pong signal at position 10 can be observed when the highly abundant piRNAs at positions 1162 and 1164 are excluded (fig. 2). The amount of piRNA in the cold invasion at generation 22 was not sufficient for computing ping-pong signatures. In both the hot and the cold invasion the height of the peak at position 10 (*h*) was weakest in the earliest generation for which the signature could be computed (hot ${\bar{h}}_{g22}=0.17$, ${\bar{h}}_{g108}=0.40$, Welch Two Sample *t*-test p=0.008; cold ${\bar{h}}_{g54}=0.17$, ${\bar{h}}_{g79}=0.21$, Welch Two Sample *t*-test p=0.41; fig. 2; supplementary fig. S11, Supplementary Material online). Also the ping-pong *z*-score, which indicates the significance of the ping-pong signature, is lower at the early generations, albeit less significantly than *h* (hot ${\bar{z}}_{g22}=4.37$, ${\bar{z}}_{g108}=16.49$, Welch Two Sample *t*-test p=0.065; cold ${\bar{z}}_{g54}=5.27$, ${\bar{z}}_{g79}=5.76$, Welch Two Sample *t*-test p=0.86; fig. 2; supplementary fig. S11, Supplementary Material online). We observed clear ping-pong signatures mostly after the P-element copy number stabilized. This suggests that the secondary piRNAs are not the main source of piRNAs at the beginning of the plateauing phase. We propose that the ping-pong cycle may be more important for silencing of the P-element at later generations.

Slightly less piRNA was observed at the plateau of the cold invasions than at the plateau of the hot invasions (fig. 1*C*; average piRNAs in ppm, hot generations 22–108 r1=2723, r3=2229, r5=3331; cold generations 70–79 r1=2193, r3=1994, r5=3279). This is surprising given that the P-element expression is higher at hot than at cold conditions (Kofler et al. 2018) (copy numbers at the plateau are similar at both conditions). This suggests that either more piRNAs are produced than required for silencing or that piRNAs silence more efficiently at hot conditions.

Even when a TE is silenced by the piRNA pathway and the copy number remains stable, it remains an open question whether the TE is silenced in all individuals of a population or if the TE remains active in a small subset in the population. Gonadal dysgenesis (GD) estimates the fraction of females with atrophied ovaries. GD is a consequence of a high P-element activity (Moon et al. 2018) in females with functional P-elements but insufficient amounts of piRNAs to repress the P-element (Brennecke et al. 2008; Kelleher 2016). In our GD assays of populations with a plateauing P-element copy numbers, a fraction of the females showed GD. This suggests that within populations silencing of the P-element is not complete and in some individuals the P-element may remain highly active. At generation 57 and 62 between 12–17% of the ovaries are dysgenic in replicates 1 and 3. At generation 79 only 0.0–1.2% of the ovaries were dysgenic in these two replicates, which confirms that the cold invasion is mostly silenced at generation 70. Interestingly, in these two replicates the difference in atrophied ovaries between generation 62 and 79 is only to a minor extent reflected by the levels of piRNA (fig. 1*C* and *D*), but by an increase of the ping-pong signature (fig. 2). Since we measured piRNAs levels from pooled flies, but estimated GD in individual females, our results suggest that the distribution of piRNAs differs among individual females. This may reflect the segregation of different cluster insertions in the evolving populations. At later generations, the cluster insertions may be more homogeneously distributed among individual flies, resulting in more females without GD. An alternative interpretation is that the production of the initial piRNAs against an invading TE is unstable with stochastic fluctuations in piRNA levels. Only when the ping-pong cycle is fully established, a robust piRNA silencing is achieved across all females.

If the trap model holds, P-element insertions in piRNA clusters should emerge during the invasion, especially at the plateau of the invasions (Kofler 2019). Therefore, we determined the positions of piRNA clusters in a long-read assembly of *D. simulans* (Chakraborty et al. 2021). We aligned all small-RNA data from the cold invasion to the genome and identified regions with a high density of piRNAs (i.e., piRNA clusters) using a previously described algorithm that maximizes a local-score (Fariello et al. 2017; Kofler et al. 2018). The piRNA clusters in *D. simulans* have a total size of 4.24 Mb which corresponds to about 2.6% of the genome (assuming a genome size of 162 Mb for *D. simulans*; Bosco et al. 2007). The positions of P-element insertions were inferred from the Pool-Seq data using PoPoolationTE2 (Kofler et al. 2016). The first P-element insertions in piRNA cluster were observed in all replicates at generation 50 of the cold invasion (supplementary figs. S13–S15, Supplementary Material online).

Next we asked if the observed number of cluster insertions is sufficient to stop an invasion under the trap model. Even under the assumption that one cluster insertion per diploid individual is biochemically sufficient to silence a TE, the number of cluster insertions required to stop an invasion in a population will be higher when cluster insertions are segregating as predicted by the trap model (Kelleher et al. 2018; Kofler 2019). Segregation of cluster insertions leads to a distribution of cluster insertions in a population where some individuals will end up with many cluster insertions while others will end up with fewer or even without any cluster insertion. The TE will be activated in individuals without cluster insertions and thus the number of cluster insertions will increase until a sufficiently high average number is achieved (Kofler 2019). When diploid individuals carry on the average four cluster insertions, the vast majority of the individuals within a population will end up with at least one cluster insertion (hence the TE is largely silenced throughout the population). Only if a cluster insertion is fixed, two insertions per diploid individual (i.e., a homozygous insertion) are sufficient to silence a TE (Kofler 2019). We found that all cluster insertions in the cold and hot invasion are segregating at a low population frequency (supplementary figs. S13–S18, Supplementary Material online) and therefore two cluster insertions per haploid genome (i.e., four per diploid) are required to stop the invasion in our experimental populations.

Using stringent criteria (unambiguously mapped piRNAs to define the location of piRNA clusters and unambiguously mapped reads to locate TE insertions), the mean number of cluster insertions per haploid genome was 0.21 at the plateau of the cold invasion (generations 70–100) and 0.24 at the plateau of the hot invasion (generation 20–60; supplementary table S6, Supplementary Material online), which is insufficient to stop an invasion under the trap model.

One possible explanation for the low number of cluster insertions may come from telomere-associated sequences (TAS) (Zhang et al. 2020). TAS are located at the ends of the major chromosome arms in *Drosophila* and likely act as piRNA clusters (Asif-Laidin et al. 2017). Since TAS largely consist of repetitive sequences, it is difficult to identify TE insertions based on unambiguously aligned reads in these regions (Zhang et al. 2020). To find TE insertions in TAS we first extracted the regions between the most distal gene and the end of each chromosome arm, yielding in total five sequences of TAS regions (X-TAS, 2L-TAS, 2R-TAS, 3L-TAS, 3R-TAS; for coordinates, see supplementary table S7, Supplementary Material online). From our paired-end reads, we additionally extracted all reads where the mate, but not the focal read, maps to the P-element. These reads enabled us to anchor P-element insertions in a reference sequence (henceforth “anchor reads”). Alignment of these anchor reads to the five TAS sequences allowing for ambiguously mapped reads showed that P-element insertions are indeed highly enriched in TAS (5.9-fold at the plateau of the cold invasion; supplementary table S8, Supplementary Material online). This enrichment was most pronounced in X-TAS followed by 3R-TAS (X=16.7, 3R=10.1; supplementary table S8, Supplementary Material online). This is consistent with previous works showing that P-element insertions in X-TAS are abundant and likely involved in silencing the P-element in *D. melanogaster* (Ajioka and Eanes 1989; Ronsseray et al. 1991; Karpen and Spradling 1992; Marin et al. 2000; Zhang et al. 2020). The enrichment of P-element insertions in TAS was more pronounced at hot than at cold conditions (hot generation 60: all=14.6, X=39.3, 3R=23.5; cold generation 100: all=5.3, X=15.0, 3R=11.0; Welch Two Sample *t*-test $p_{\mathrm{all}}=0.06$, $p_{X}=0.07$, $p_{3R}=0.10$; supplementary table S8, Supplementary Material online). Nevertheless, even when TAS insertions are considered, the average number of insertions per haploid genome is still not sufficient to stop the P-element invasions under the trap model (cold=0.37, hot=0.64; not counting TAS insertions twice; supplementary table S6, Supplementary Material online).

Under the most liberal criteria where we consider (i) TAS regions (ii) ambiguous mapping of piRNAs and and TE insertions and (iii) polymorphism of piRNA clusters we estimate that each individual could carry about 1.58 cluster/TAS insertions at cold and 1.81 at hot conditions (supplementary results S1, Supplementary Material online).

To summarize we estimate that at the plateau of the invasion each individual carries on the average between 0.21 (stringent criteria) and 1.81 (most liberal criteria) cluster insertions per haploid genome, which is smaller than the 2 insertions expected under the trap model (without paramutations; see Discussion). Although piRNAs against the P-element and P-element insertions in piRNA clusters rapidly emerged in our experimental populations the number of cluster insertions is probably not sufficient to control an invasion under the trap model.

### Influence of Temperature on Factors that Might Influence Invasion Dynamics

We showed that the transposition rate had little influence on P-element copy numbers at the plateau of an invasion. Since differences in transposition rate were modulated by temperature it is important to test whether temperature affects other factors influencing the invasion dynamics of the P-element.

**Fig. 3.**
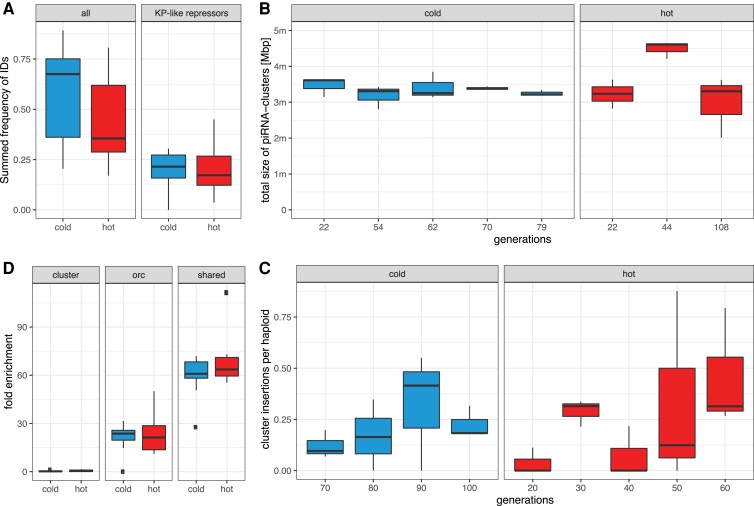
Temperature has a minor influence on some important factors governing the invasion dynamics. (*A*) Fraction of P-element insertions with any internal deletion (ID; all) and an ID that may repress P-element activity (KP-like repressor). Only samples at the plateau of the invasions were used (hot ≥20, cold ≥70 generations). (*B*) Total size of piRNA clusters during the invasions based on unambiguously mapped piRNAs. (*C*) Average number of P-element insertions in piRNA clusters per haploid genome. Only samples at the plateau of the invasion are shown. (*D*) Enrichment of P-element insertions in piRNA clusters, ORCs and insertion sites shared among species. Only samples with more than 10 P-element insertions per haploid genome were used (generations ≥20 hot and ≥50 cold).

We first investigated the influence of internal deletions (IDs) of the P-element since IDs may repress P-element activity and our previous work showed that IDs emerge faster at hot than at cold conditions (Kofler et al. 2018). IDs, such as KP or D50 (Black et al. 1987; Rasmusson et al. 1993) produce non-functional transposases that retain the DNA-binding capacity but are unable to mobilize the P-element. KP-like repressors prevent functional transposases from mobilizing the P-element by blocking transposase binding sites (supplementary fig. S19*A*, Supplementary Material online, green shade in lower panel). We measured the dynamics of IDs in hot and cold condition using DeviaTE allowing for 3 bp tolerance in the position of IDs (the exact position of ID is frequently ambiguous in alignments). We found 65 distinct IDs in the cold invasion and 61 in the hot invasion. Nine IDs shared the same break points in multiple replicates. Assuming that identical break points rarely arise independently, we conclude that these IDs were already present in the base population (7 out of the 9 IDs were also detected in the cold invasion; supplementary fig. S19*A*, Supplementary Material online, dashed lines). Some IDs increased in frequency within the P-element population (supplementary fig. S19*B*, Supplementary Material online). This frequency increase may be driven either by positive selection resulting from the repression of the P-element activity or by preferential mobilization compared to other P-elements. Especially, the ID at position 187–1967 nt was rapidly rising in frequency in two replicates, accounting for up to 30–40% of all P-element copies at the last generation (supplementary fig. S19*B*, Supplementary Material online, red). This ID is probably no KP-like repressor as its DNA-binding domain is largely deleted (supplementary fig. S19, Supplementary Material online, red; orange shade in lower panel A is the DNA-binding domain). This makes positive selection unlikely. Rather, preferential mobilization of this ID is a more plausible explanation for the copy number increase of this ID. Although IDs are emerging faster at hot than at cold conditions (Kofler et al. 2018), the final abundance of IDs and KP-like repressors is similar at the two temperature conditions (fig. 3*A*; Welch Two Sample *t*-test hot generation 60 vs. cold generation 100; all IDs p=0.34; KP-like repressors p=0.57). It is thus also unlikely that P-elements are differently regulated by IDs in the two temperature regimes.

Simulations of TE invasions with piRNA clusters showed that the size of piRNA clusters is the major factor determining the plateauing level of TE invasions (Kelleher et al. 2018; Kofler 2020). As the activation of some piRNAs cluster may depend on temperature (Casier et al. 2019) we tested whether differences in the size of piRNA clusters could have influenced the invasion dynamics. We estimated the size of piRNA clusters in our small RNA data based on unambiguously mapped piRNAs. The size of the piRNA cluster is very similar for hot and cold conditions (Welch Two Sample *t*-test with average size of piRNA cluster per replicate p=0.45; fig. 3*B*).

Additionally, the abundance of P-element insertions in piRNA clusters identified using small-RNA data (stringent conditions, with unambiguously mapped piRNAs and TE insertions) is also similar in the two temperature regimes—irrespective of whether piRNAs from the hot or cold conditions were used to identify piRNA clusters (Welch Two Sample *t*-test with average number of cluster insertions per replicate; cold piRNAs, p=0.45; fig. 3*C*; hot piRNAs, Welch Two Sample *t*-test; p=0.96).

It has been suggested that the P-element has an insertion bias into diverse genomic features such as promoters of genes, TAS and origin of replication complexes (ORC) (Karpen and Spradling 1992; Bellen et al. 2011; Spradling et al. 2011; Kofler, Hill, et al. 2015). These insertion biases may enhance (e.g., ORC) or counteract (e.g., piRNA clusters including TAS) the propagation of the P-element. Since differences in the insertion bias among hot and cold conditions could influence invasion dynamics we investigated the extent of this bias for (i) piRNA clusters (ii) ORCs and (iii) insertion sites shared between *D. melanogaster* and *D. simulans*.

We showed that the P-element has a stronger insertion bias into TAS at hot than at cold conditions. This does not necessarily imply that an invasion is more readily silenced at hot than at cold conditions, since any insertion in a piRNA cluster (not just in TAS) may trigger the production of piRNAs that silence the P-element. Therefore we tested whether the insertion bias into any piRNA cluster differs between the two temperatures (including TAS regions; stringent conditions, with unambiguously mapped piRNAs and TE insertions). However, the overall insertion bias into piRNA clusters was similar between hot and cold conditions (mean enrichment for samples with more than 10 insertions: hot=0.63, cold=0.33; Welch Two Sample *t*-test with the average enrichment per replicate p=0.33; fig. 3*D*). At both temperatures, the insertion bias was <1, implying that P-element insertions in piRNA clusters are actually less abundant than expected by chance.

An insertion bias into origin of replication complex (ORC) regions may enhance the transmission of the P-element into the next generation by enabling the P-element to be duplicated multiple times during S-phase (Spradling et al. 2011). We found a strong enrichment of P-element insertions in ORC regions at both temperatures. The extent of the enrichment was very similar between hot and cold conditions (mean enrichment for samples with more than 10 insertions: hot=23.2, cold=21.9; Welch Two Sample *t*-test with the average enrichment per replicate p=0.71; fig. 3*D*).

We previously reported that some insertion sites of the P-element are shared between *D. simulans* and *D. melanogaster* (Kofler, Hill, et al. 2015), suggesting a strong insertion bias in both species. It is not clear if this insertion bias into shared sites influences the propagation of the P-element. The enrichment at these sites is slightly higher at hot than at cold conditions (mean enrichment for samples with more than 10 insertions: hot=70.2, cold=60.6; Welch Two Sample *t*-test with the average enrichment per replicate p=0.025; fig. 3*D*). Taken together, the insertion bias is thus similar at both temperatures.

Genetic drift is a decisive factor that may influence the invasion dynamics. Negative selection against TEs (*x*) or transposition (*u*) will only be stronger than drift if the conditions $N_{e}\times x>1$ or $N_{e}\times u>1$, are met (Gillespie 2010; Kofler 2019). Differences in the extent of genetic drift between the hot and the cold invasion could therefore have a pronounced influence on the invasion dynamics. However, estimates of the effective population size during the experiment are similar among the two temperature regimes (supplementary table S9, Supplementary Material online).

It is also possible that different experimental conditions trigger heterogeneous selection pressure against P-element insertions. Rather than attempting to test each of the possible factors resulting in temperature-specific selection against TE insertions, we evaluated the insertion pattern in functionally diverged genomic regions. We estimated the proportion of P-element insertions in intergenic regions, $5^{\prime }$-UTRs, CDS, introns, $3^{\prime }$-UTRs, pseudogenes, and repeat regions. We did not observe significant differences in the distribution of P-element insertions in the two temperature regimes (supplementary fig. S20, Supplementary Material online). This analysis does, however, not exclude differences in ectopic recombination rates or transposase activity between the two temperature regimes (Kaufman and Rio 1992).

We conclude that different transposition rates are the best explanation for the temperature-specific invasion dynamics as the investigated factors (size of piRNA clusters, abundance of KP-like repressors, insertion bias, genetic drift) were similar between the temperature regimes. Also the distribution of P-element insertions in different functional genomic regions was similar in the hot and cold temperature regime.

### Modeling the P-Element Invasion

Finally, we evaluated whether the invasion dynamics of the P-element in our experimental populations could be quantitatively captured by a simple model. The trap model provides a good framework to predict the invasion dynamics when the key parameters insertion rate and piRNA cluster size are known. The trap model is a non-equilibrium model (except for unlikely scenarios, such as the TSC-balance Kofler 2019). This contrasts another widely used model, which assumes an equilibrium between TE copy numbers gained by transposition and lost by negative selection (Charlesworth and Charlesworth 1983; Charlesworth and Langley 1989). We used a constant transposition rate, which was estimated from the data before piRNAs became abundant and caused the plateauing of P-element copy numbers ($u_{\mathrm{cold}}=0.051$, $u_{\mathrm{hot}}=0.154$; i.e., the average *u*; supplementary table S2, Supplementary Material online). Initially, we assumed no residual activity of the P-element in individuals with a cluster insertion. We simulated five chromosome arms of 32.4 Mb and a piRNA cluster of size 850 kb at the end of each chromosome arm, resulting in a total genome size of 162 Mb and a total size of piRNA clusters of 4.25 Mb. These conditions resemble our observations in *D. simulans* where piRNA clusters also account for 2.6% of the genome. Additionally, we used a recombination rate of 4 cM/Mb (Howie et al. 2019), a population size of N=230, and triggered the invasions by randomly distributing 396 TE insertions with a population frequency of 1/2×N in the initial population (similarly to our base population).

Simulations with neutral TE insertions (x=0; hence $u^{\prime }=u$) capture the sharp increase in TE copy numbers but fail to predict the timing and the level of the plateauing (supplementary fig. S21, Supplementary Material online). Both the observed onset and level of the plateauing (hot 15.1 copies, 20 generations; cold 15.2 copies, 70 generations) were substantially overestimated by the simulations (hot 75 copies, ≈120–240 generations; cold 53 copies, ≈120–240 generations; supplementary fig. S21, Supplementary Material online). Furthermore, the number of P-element insertions per haploid genome was slightly lower in the cold than in the hot simulation.

We performed additional simulations to rule out that this discrepancy can be explained by underestimating the size of piRNA clusters. We show that a plateauing at about 15 insertions per haploid genome is only expected when the piRNA clusters account for about 20% of the genome (supplementary fig. S22, Supplementary Material online). Typical estimates for the size of piRNA clusters range from 3% in *Drosophila* to about 0.1% in humans, mice, and rats (Girard et al. 2006; Brennecke et al. 2007), which suggests that the piRNA cluster size cannot explain the discrepancy to the observed data.

**Fig. 4.**
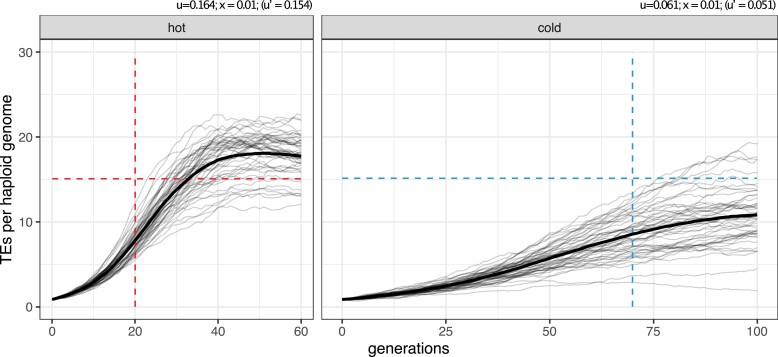
Expected and observed invasion dynamics under the trap model with negative selection against TE insertions (x=0.01). The dashed lines indicate the observed onset and level of the plateau in our experimental populations. Solid lines show the simulated invasion dynamics for 50 replicates and the bold lines are averages. *u* transposition rate, *x* negative effect of a TE insertion, $u^{\prime }$ effective transposition rate.

We considered a more complex trap model, which also accounts for selection against TE insertions. To avoid an equilibrium state between transposition, selection, and piRNA clusters (TSC-balance), we assumed that cluster insertions are neutral (Kofler 2019). In the presence of purifying selection, we cannot observe the transposition rate directly, but rather the effective transposition rate ($u^{\prime }=u-x$) which is the net result of transposition (*u*) and negative selection against TEs (*x*). The same effective transposition rate may be achieved by many different combinations of transposition rates and negative effects against TEs. Therefore, we performed simulations of TE invasion with different combinations of these two factors. The strength of negative selection had indeed a pronounced influence on the invasion dynamics, even when the effective transposition rate was kept constant (supplementary fig. S23, Supplementary Material online). A reasonable fit between expected and observed data was obtained when the negative effect of TE insertions was about x=0.01, that is, when a single TE insertion reduced host fitness on the average by 1% (supplementary fig. S23, Supplementary Material online). Under this model the onset and level of the plateauing (hot 17.3 copies, ≈40 generations; cold 10.8 copies, ≈100–120 generations) approach the observed ones (hot 15.1 copies, 20 generations; cold 15.2 copies, 70 generations; fig. 4; for more generations, see supplementary fig. S24, Supplementary Material online; panel $u_{r}=0$). Although this simple model roughly captures the dynamics of the P-element invasion, some differences still remain—the delayed plateauing in the simulations, the overshooting in the hot environment and the underestimation of the plateauing copy number in the cold environment.

We caution, however, that many open questions remain about factors influencing invasion dynamics and therefore other models, for example, using other combinations of parameters, may fit the data equally well.

## Discussion

We studied the influence of transposition rates on the invasion dynamics of the P-element. We show that different transposition rates, modulated by the ambient temperature of experimental *D. simulans* populations, have little influence on the number of TE insertions at the plateau. Since temperature may not only affect the transposition rates, but also other factors, we evaluated a broad range of factors (IDs, insertion bias, effective population size, size of piRNA clusters) and only found a higher insertion bias into TAS regions, especially X-TAS, at hot than at cold conditions.

A preferential insertion into TAS regions at hot conditions might lead to a faster silencing of the invasion and thus a lower plateau level at hot conditions. However, since the TAS regions are a subset of the piRNA producing loci and the overall insertion bias into any piRNA producing locus is similar among the two temperatures (fig. 3*D*), similar plateauing levels at the two temperatures are expected. In agreement with this, the hot and the cold invasions plateaued at a very similar level in our experimental populations (cold 12–18 insertions, hot 13–17 insertions; fig. 1*A*).

Gonadal dysgenesis is highly temperature-dependent. At high temperatures, GD is very strong (≈29°C), but below 24°C it is rarely observed (Engels and Preston 1979; Kidwell and Novy 1979; Ghanim et al. 2020). It is assumed that GD slows the spread of the P-element (Kelleher et al. 2018), but the impact of negative selection against dysgenic females is not entirely clear. GD will reduce the fitness of males having too many P-element insertions (they induce GD) as well as of females having too few P-element insertions (they are not producing piRNAs and are thus susceptible to P-element activity). Furthermore, GD within populations is probably a transient phenomenon (expected during the early stages of an invasion), that can be attributed to initially low levels of piRNAs. To shed more light on the impact of GD during our experiment, we previously measured GD at the experimental temperatures and did not observe GD in the hot or cold invasions (Kofler et al. 2018). GD was only observed at temperatures even higher than experienced during the experimental evolution (29°C) (Kofler et al. 2018). While these results suggest that GD is unlikely to affect the dynamics of our invasion, we cannot fully rule out that GD had a weak effect at hot conditions.

The P-element invasion in our experimental *D. simulans* populations plateaued at very similar levels. This raises the question whether P-element invasions in natural populations also plateau at similar copy numbers. The number of P-element insertions varies among different *D. melanogaster* strains and populations (Ronsseray et al. 1989; Biémont et al. 1990; Bergman et al. 2017; Weilguny et al. 2020). One study found copy number differences as extreme as 10 and 80 insertions per haploid genome among populations collected in the wild (Weilguny et al. 2020). Such differences in P-element copy number among populations can have multiple reasons. First the P-element invasion might not yet have plateaued in some local *D. melanogaster* populations. Second the abundance of repressors of P-element activity, such as the KP-element (Black et al. 1987) differs among populations (Bergman et al. 2017; Weilguny et al. 2020) which could have an effect on the plateauing level. Third, since piRNA clusters are evolving rapidly (Gebert et al. 2021; Wierzbicki et al. 2021) the size of piRNA clusters may differ among local populations, which would have a major impact on the plateauing level (Kofler 2019). Fourth the genetic background will differ among populations and this could, for example, influence genes that regulate the tolerance of the host to P-element activity [e.g., *bruno* (Kelleher et al. 2018)].

S imulations of the P-element invasion showed that a trap model with piRNA clusters accounting for about 2.6% of a genome and deleterious TE insertions (x=0.01) roughly captures the invasion dynamics of the P-element (fig. 4). However, this model rests on several assumptions that may be violated.

First we assumed that a TE invasion is stopped when the TE jumps into a piRNA cluster (i.e., the trap model). While there is ample support for this model (Josse et al. 2007; Muerdter et al. 2012; Zanni et al. 2013; Duc et al. 2019; Kofler 2019) some doubts recently emerged (Yu et al. 2019; Gebert et al. 2021). Notably, deletion of the three largest piRNA clusters did not lead to an increase of TE activity (Gebert et al. 2021). The authors argue that instead of cluster insertions, dispersed piRNA producing TE insertions are responsible for maintaining silencing of TEs. Such dispersed piRNA producing TE insertions may be the result of paramutations, where maternally transmitted piRNAs mediate the conversion of dispersed TE insertions into piRNA-producing loci (de Vanssay et al. 2012; Le Thomas et al. 2014; Mohn et al. 2014; Hermant et al. 2015). Notably, the origin of the initial piRNAs against an invading TE is unclear under this scenario (Gebert et al. 2021). One possibility is that the production of the initial piRNAs is triggered by an insertion into a piRNA cluster. Paramutations could have a substantial effect on invasion dynamics. The emergence of some initial piRNAs against the invading TE (possibly due to a cluster insertion) may trigger a chain-reaction, where increasing numbers of euchromatic TE insertions are converted into piRNA producing loci. In the absence of negative selection against TEs, such an accelerated silencing could account for the rapid onset of the plateauing and the low abundance of the P-elements at the plateau in our experimental populations. If paramutations contribute to the silencing of P-elements, not every individual in a population needs to carry piRNA cluster insertions as euchromatic TE insertions may also produce piRNAs. Paramutations may thus also account for the low number of cluster insertions found in our experimental populations. The influence of paramutations and piRNA cluster insertions on invasion dynamics remains an important open question.

Second we assumed that the emergence of piRNAs against an invading TE leads to complete silencing of a TE reducing the transposition rate to zero. It is however feasible that TEs controlled by the piRNA pathway retain some residual activity (u>0) which could have a notable effect on the invasion dynamics. The slight increase in P-element abundance between generations 90–100 of the cold invasion suggests that the P-element may have some residual activity, despite being largely silenced by the piRNA pathway. It is possible that previous estimates of the transposition rates for many TE families represent the transposition rate of TEs being silenced by the piRNA pathway (ranging from $1.2\times 10^{-5}$ to $5\times 10^{-4}$  Nuzhdin and Mackay 1995). Our computer simulations show, however, that a small residual activity has little impact on the invasion dynamics (supplementary fig. S24, Supplementary Material online).

Third our estimates of the effective population size range between 179 and 297 for different replicates and temperatures, whereas our simulations were performed with the average of N=230 (supplementary table S9, Supplementary Material online). Additional simulations with effective populations sizes ranging from 100 to 300 produce highly similar invasion dynamics (supplementary fig. S25, Supplementary Material online), indicating that the experimental variation in population size does not explain the observed differences in invasion dynamics.

Fourth we used a very simple selection model. The assumed fitness cost for a P-element insertion of 1% (x=0.01) is lower than the 5.5% estimated by Mackay et al. (1991) for a heterozygous P-element insertion in *D. melanogaster*. We further assumed that all TE insertions have the same effect on host fitness. However, it is likely that the fitness consequence of TE insertions varies across the genome; for example insertions within important genes are more deleterious than insertions in repetitive regions (Petrov et al. 2011; Kofler et al. 2012). Interestingly, our simulations show that the distribution of fitness effects has little influence on the onset and the level of plateauing, but a marked influence on the TE abundance after the plateauing (supplementary fig. S26, Supplementary Material online). With equal fitness effects of all TE insertions, for example, our model predicts a sharp decline in TE copy numbers at later generations. While we currently lack sufficiently advanced generations to test this prediction against empirical data, nearly stable copy numbers are obtained when the negative effects of the TEs are drawn from a distribution with a mean effect of $\bar{x}=0.01$ (supplementary fig. S26, Supplementary Material online; equal amounts of insertions with x∈{0.0,0.005,0.01,0.015,0.02}).

We assumed that fitness decreases linearly with TE copy numbers but it is unclear if this assumption holds. It is feasible that fitness decreases exponentially with TE copy numbers (Charlesworth and Charlesworth 1983), for example mediated by the deleterious effects of ectopic recombination (Charlesworth and Langley 1989; Montgomery et al. 1991). It remains an important open question whether or not ectopic recombination contributes to limiting the spread of TEs (Barrón et al. 2014; Kent et al. 2017). Finally, it is conceivable that the extent of negative selection against TE insertions differs among the temperatures. Such differences could, for example, emerge when an active transposase is generating harmful cellular effects (Nuzhdin et al. 1998) and the activity of the transposase depends on the temperature (Kaufman and Rio 1992). These considerations highlight that the distribution of fitness effects of TE insertions is a crucial factor determining invasion dynamics. Unfortunately, little is known about the fitness effects of TEs. Although many open questions remain about important parameters, modeling of TE invasions is an important step towards a quantitative understanding of TE invasions. Such a quantitative understanding might finally shed light on the question, why the TE composition varies so dramatically among species (Biémont and Vieira 2006; Feschotte and Pritham 2007).

## Materials and Methods

### Experimental Populations

Experimental populations were maintained as described previously (Kofler et al. 2018). Briefly, we collected 202 isofemale lines from a *D. simulans* population collected in November 2010 in Florida (Tallahassee). To mimic the natural day–night cycle of *Drosophila*, the temperature cycled between two fixed temperatures every 12 h. We established three replicate populations that were exposed to hot (cycling between 18°C and 28°C) and cold (cycling between 10°C and 20°C) conditions. The census population size was about N=1000−1250 and non-overlapping generations were used.

### Genomic Sequencing Data

We previously sequenced the hot evolved populations until generation 60 and the cold evolved populations until generation 40. All populations were sequenced as pools (Pool-Seq Schlötterer et al. 2014) at each 10th generation using the Illumina paired-end technology (Accession numbers PRJEB20533 and PRJEB20780).

Here we additionally sequenced the cold evolved populations at each 10th generation from generation 50 to 100. For each sample the DNA of pooled female and male flies was extracted using a high salt extraction protocol (Miller et al. 1988) and sheared with a Covaris S2 device (Covaris, Inc. Woburn, MA, USA). Libraries were prepared using the TruSeq DNA PCR-Free protocol (Illumina, San Diego, CA) and sequencing was performed with the Illumina HiSeq X Ten platform (Illumina, San Diego, CA). For an overview of the sequencing data used in this work see supplementary table S1, Supplementary Material online.

### small RNA

In addition to previously published small RNA data (hot at generations 22, 44, 108; cold at generations 22, 54; Kofler et al. 2018) we sequenced small RNA from cold evolved populations at generations 62, 70, 79. As described previously we extracted total RNA from about 50 females (aged 3–4 days) reared in a common garden for two generations at 23°C. Sequencing of the small RNA libraries was done by Fasteris (https://www.fasteris.com/dna/). To avoid inconsistencies, all small RNA data, including the previously published ones, were analyzed using the same pipeline. We initially filtered for reads with a length between 15 and 35 bp and aligned them to a database consisting of the *D. simulans* tRNAs, miRNAs, mRNAs, snRNAs, snoRNAs, rRNAs (v1.4; Flybase http://flybase.org/) as well as the consensus sequences of TEs in *D. melanogaster* [v9.42; plus Mariner GenBank:M14653.1 (Quesneville et al. 2005)] using novoalign (v3.03.02; http://www.novocraft.com/ -F STDFQ -o SAM -o FullNW -r RANDOM). We filtered small RNAs with more than two mismatches. The abundance of different small RNAs, the distribution of piRNAs within the P-element, the length distribution of the piRNAs and the ping-pong signal were computed with previously described Python scripts (Kofler et al. 2018). The *z*-scores were computed as $z=(h_{10}-\mu_{!10})/\sigma_{!10}$, where $h_{10}$ is the height of the ping-pong peak at position 10, $\mu_{!10}$ the mean height of the peaks from position 1 to 20 excluding position 10 and $\sigma_{!10}$ the standard deviation of these peaks. For an overview of the composition of all small RNA samples, see supplementary table S3, Supplementary Material online.

To identify the positions of piRNA clusters, we aligned small RNA reads with a length between 23 and 29 bp to the reference genome of *D. simulans* [strain $w^{XD1}$ (Chakraborty et al. 2021)], filtered for uniquely mapping reads (mapping quality >0), counted the number of reads for genomic bins of 500 bp, normalized the abundance to a million mapped miRNAs, and clustered bins with a high number of piRNAs using a previously described algorithm that maximizes a local score (Fariello et al. 2017; Kofler et al. 2018) (*localscore-pirnaclusters.py* --threshold 10 --binsize 500 --max-bin-score 100)

### Gonadal Dysgenesis

In addition to the previously published gonadal dysgenesis assays of the cold invasion at generation 57 (Kofler et al. 2018), we performed gonadal dysgenesis assays at generations 62, 71, and 79 for this work. Gonadal dysgenesis assays were performed as described previously (Kofler et al. 2018). As GD is stronger in flies raised at high temperatures (Kidwell et al. 1977; Moon et al. 2018) we kept fly eggs until eclosion at a temperature of 29°C. Eclosed flies were kept for two days at a 23°C on apple juice agar with live yeast, before dissection in PBS. We used the following classification of ovaries: clearly visible ovarioles or eggs (normal), ovarioles barely visible (intermediate), ovarioles or eggs could not be detected (dysgenic). The percentage of dysgenic ovaries was computed as 100×(dysgenic+(intermediate/2))/(normal+intermediate+dysgenic)

### TE Abundance and Diversity

To avoid inconsistencies, we analyzed all Pool-Seq data, including the previously published ones, with the same pipeline. We estimated the abundance and diversity of TEs with DeviaTE (Weilguny and Kofler 2019). Short reads were aligned with bwa sw (v0.7.4 Li and Durbin 2009) to the consensus sequences of TEs in *D. melanogaster* (Quesneville et al. 2005) and the sequences of three single copy genes (*traffic jam*, *rpl32* and *rhino*). The abundance of TEs is estimated as the coverage of TEs normalized to the coverage of the single copy genes. DeviaTE also provides the position and frequency of internal deletions of the P-element.

The number of reads mapping to the P-element (rpm) and the position of P-element insertions in the genome was determined with PoPoolationTE2 (v1.10.04 Kofler et al. 2016). To increase the inner distance, which is a major factor determining the power of PoPoolationTE2, we trimmed reads to a length of 75 bp (supplementary table S1, Supplementary Material online). Reads were mapped as single-ends with bwa sw (0.7.4 Li and Durbin 2009) to a reference genome consisting of the sequence of the P-element (GenBank:X06779.1 O’Hare and Rubin 1983) and a long-read based assembly of *D. simulans* [$w^{XD1}$ (Chakraborty et al. 2021)]. Using PoPoolationTE2 we restored the paired-end information (se2pe), generated a ppileup file (*ppileup* --map-qual 15), subsampled the ppileup to a uniform coverage of 15 (*subsamplePpileup* --target-coverage 15), identified signatures of TE insertions (*identifySignatures* --mode separate, --min-count 1, --signature-window fix100), estimated the population frequencies of TE signatures (*frequency*), filtered TE signatures (*filterSignatures* --min-count 1, --max-otherte-count 2, --max-structvar-count 2) and finally paired TE signatures to obtain a list of P-element insertions (*pairupSignatures*). For estimating the transposition rate (*u*), we assumed that TE copy numbers (*n*) at a time (*t*) may be computed from initial copy numbers ($n_{k}$) as $n_{k+t}=n_{k}(1+u{)}^{t}$. The transposition rate is then: $u=-1+\sqrt{[}t]n_{k+t}/n_{k}$.

To find the positions of ORCs in *D. simulans*, we aligned the ORC sequences of *D. melanogaster* (Spradling et al. 2011) to *D. simulans* ($w^{XD1}$) with bwa sw (v0.7.17; see also Kofler et al. 2018). To identify the location of insertions sites shared between *D. melanogaster* and *D. simulans* we extracted 1,000 bp flanking the previously described insertion sites (Kofler et al. 2016) and aligned these flanking sequences to *D. simulans* ($w^{XD1}$) with bwa sw (v0.7.17). Finally, we filtered for a minimum size of 500 (for ORCs or shared regions) and merged overlapping annotations. The enrichment of P-element insertions (*e*) was computed as e=o/(f*t), where *o* is the observed number of P-element insertion in a feature (ORCs, piRNA clusters, conserved insertion sites), *f* the genomic fraction of the feature and *t* the total number of P-element insertions.

To estimate the abundance of P-element insertions in diverse genomic features—such as CDS, intron, 5-UTR—we used an annotated assembly of the *D. simulans* strain *M252* (Palmieri et al. 2015) to identify P-element insertions with PoPoolationTE2 as described above. The proportion of insertions in given genomic features was estimated using a custom script (annotation−statistic.py).

### Telomeric-Associated Sequences

To identify the position of TAS, we extracted the sequences of all genes of *D. melanogaster* (v6.39; https://flybase.org/) and *D. simulans* (v2.02; https://flybase.org/) into a fasta file and aligned them to the reference genome of *D. simulans* ($w^{XD1}$) with bwa sw (v0.7.17). We annotated the sequences between the last genes (either from *D. melanogaster* or *D. simulans*) and the telomeric ends of the chromosome arms as TAS (supplementary table S7, Supplementary Material online). To obtain the sequences of anchor reads, that is, reads where solely the mate but not the focal read maps to the P-element, we aligned all Pool-Seq data to the sequence of the P-element (GenBank: X06779.1) with bwa sw (v0.7.4) and extracted the anchor reads using a custom script (bam2pelematefastq.py). Finally, the anchor reads were aligned to the TAS sequences, sequences of piRNA clusters or the assembly of *D. simulans* ($w^{XD1}$) using bwa sw (v0.7.17).

### Genetic Drift

The effective population size was estimated based on paired-end reads mapped to the *D. simulans* strain M252 (see above). We generated a pileup file with samtools [v1.2 (Li et al. 2009); mpileup -q 20 --excl-flags 0x04,0x08 -B --min-BQ 0 -d 100000 -A] and further converted the pileup into a sync file with PoPoolation2 (Kofler et al. 2011). For each experimental population, we picked 10 sets of 5,000 random SNPs distributed over the autosomes (2L, 2R, 3L, 3R, 4). We estimated the effective population size over the experiment (60 generations for hot conditions; 100 generations for cold conditions) with Nest (Jónás et al. 2016; Taus et al. 2017) and averaged the estimates among the 10 sets of random SNPs.

### Simulated TE Invasions

Simulations of TE invasions with piRNA clusters were performed with Invade (v0.8.07) (Kofler 2019). We simulated five chromosome arms of 32.4 Mb and a piRNA cluster of size 850 kb at the end of each chromosome giving a total genome size of 162MB and a total size of piRNA clusters of 4.25 Mb. We used a population size of N=230 (based on our estimate of the effective population size; supplementary table S9, Supplementary Material online) and a uniform recombination rate of r=4 cM/Mb [with a window size of 2.5 Mb the recombination rate in *D. simulans* varies largely between 2.5 and 5 cM/Mb (Howie et al. 2019)]. To trigger the invasions we randomly distributed 396 TE insertions in the population each with a population frequency of 1/2×230, which corresponds to 0.86 TE insertions per haploid genome as found in our base populations (supplementary table S2, Supplementary Material online). Negative selection against TEs was simulated with the linear fitness function w=1−xn where *x* is the negative effect of a TE insertion and *n* the number of TE insertions. Simulations were run for 1,000 generations.

## Supplementary Material

Supplementary data are available at *Molecular Biology and Evolution online*.

## Supplementary Material

msac141_Supplementary_Data

## Data Availability

All scripts used in this work are available at https://sourceforge.net/p/te-tools/code/HEAD/tree/ (folder “coldinvasion”). All data have been made publicly available at the European Nucleotide Archive (PRJEB47624).
