# Supplementary material for: The Transposition Rate Has Little Influence on the Plateauing Level of the P-element
Source: Mol Biol Evol. 2022 Jun 22;39(7):msac141. doi: 10.1093/molbev/msac141 (PMC9254008; doi:10.1093/molbev/msac141)
Supplement: msac141_Supplementary_Data [file msac141_supplementary_data.pdf]

Supplementary figures and tables

June 8, 2022

Supplementary Figures

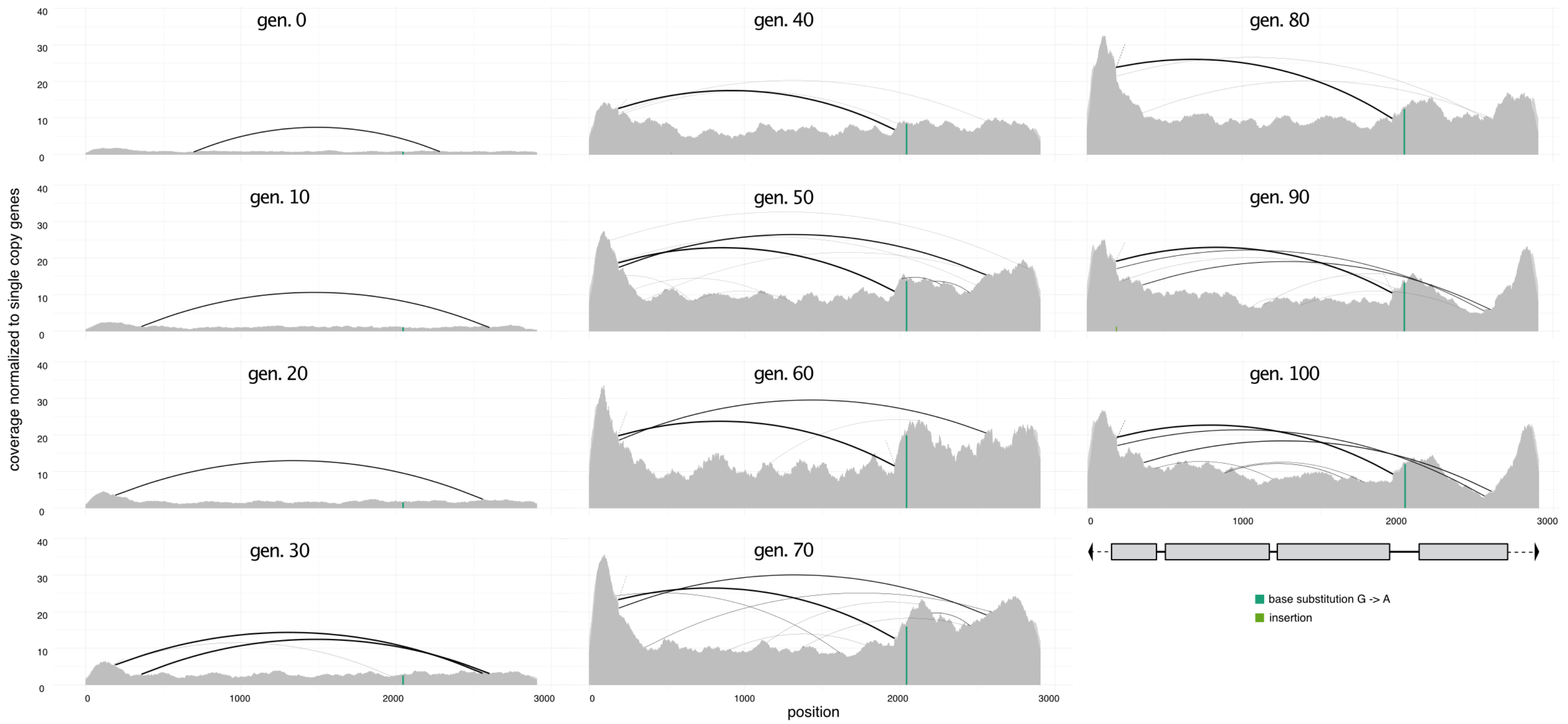

Figure 1: Abundance and diversity of the P-element during the invasion (gen.: generations) at cold conditions in replicate 1. Short reads were aligned to the consensus sequence of the P-element and visualized with DeviaTE. The coverage of the P-element was normalized to the coverage of single-copy genes. The coverage therefore reflects the number of P-element insertions (e.g. a coverage of 20 corresponds to about 20 P-element insertions). Coverage based on uniquely and ambiguously mapped reads is shown in dark and light gray, respectively (only few ambiguously mapped reads were found at the terminal ends of the P-element). Differences to the consensus sequence of the P-element in *D. melanogaster* are shown as colored lines (base substitutions and indels), where the height of the line indicates the abundance of the variant. Large internal deletions are shown as black arcs. The structure of the P-element is indicated at the end, where TIRs (black triangles), the four ORFs (grey rectangles) and introns (black lines) are shown.

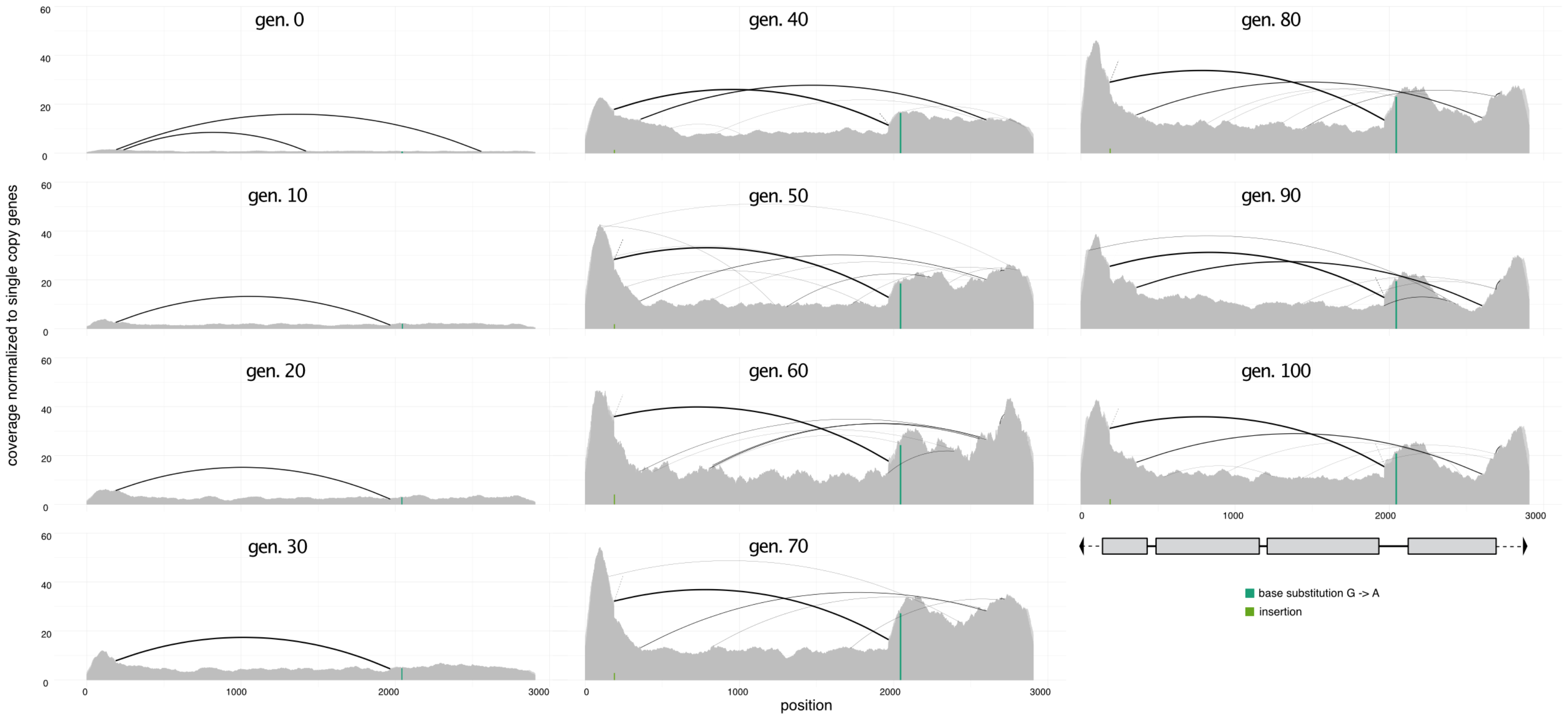

Figure 2: Abundance and diversity of the P-element during the invasion (gen.: generations) at cold conditions in replicate 3. For details see caption of supplementary fig. 1.

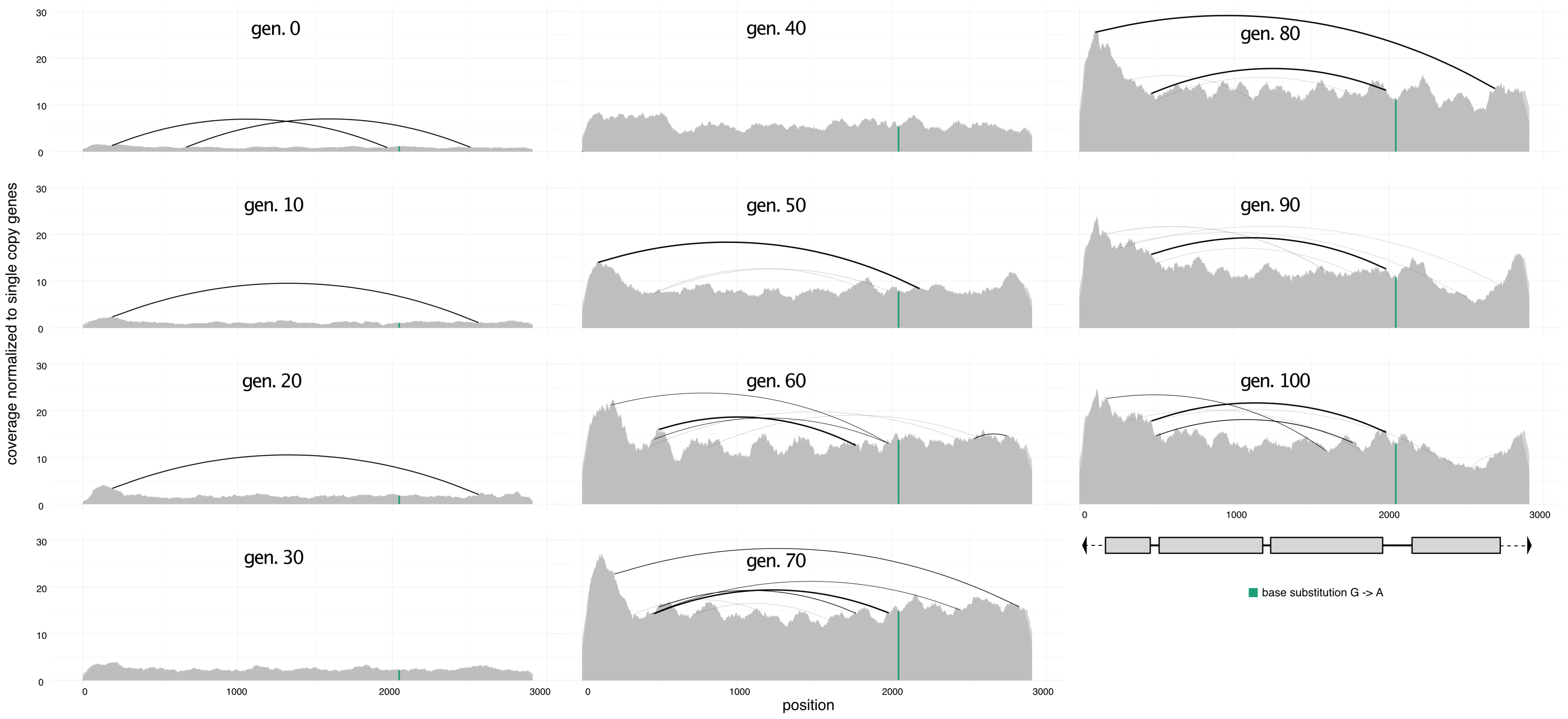

Figure 3: Abundance and diversity of the P-element during the invasion (gen.: generations) at cold conditions in replicate 5. For details see caption of supplementary fig. 1.

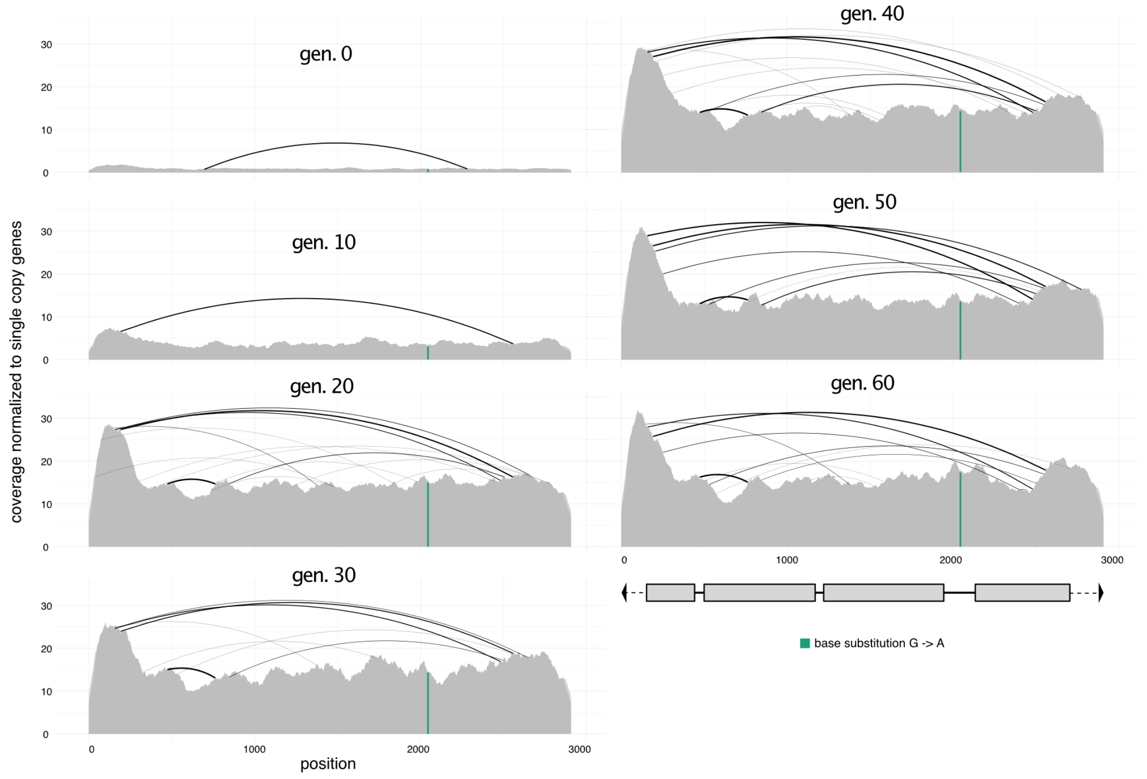

Figure 4: Abundance and diversity of the P-element during the invasion (gen.: generations) at hot conditions in replicate 1. For details see caption of supplementary fig. 1.

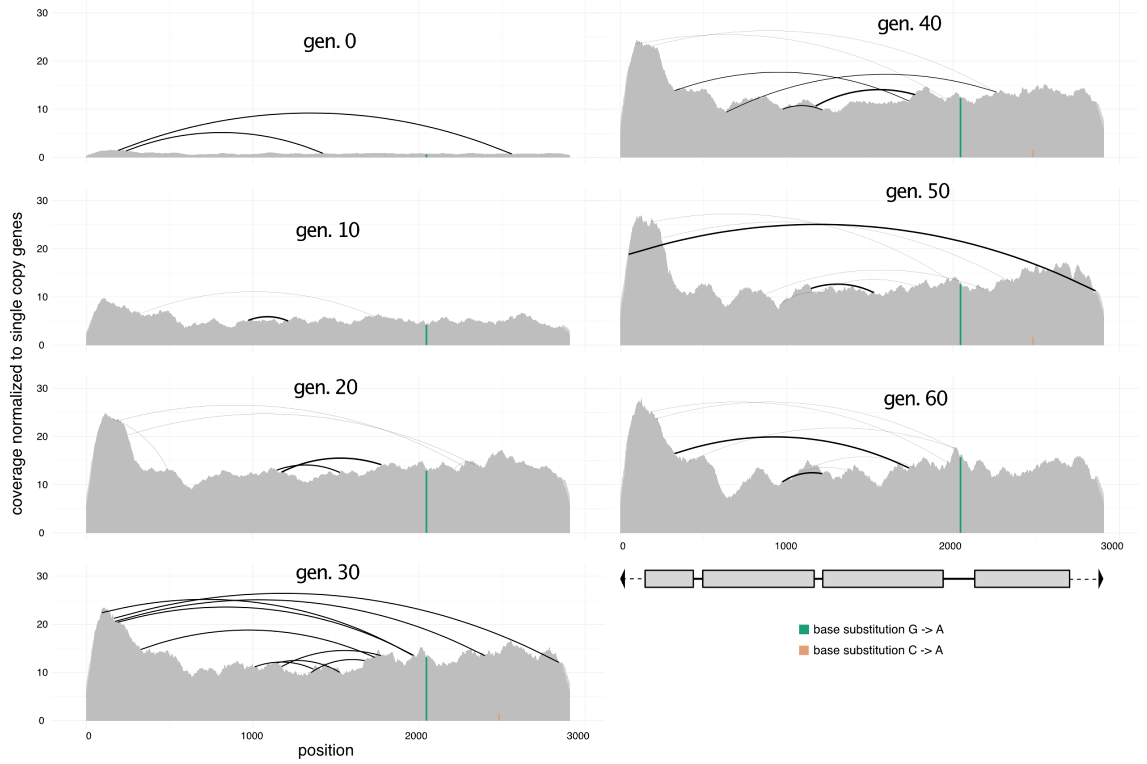

Figure 5: Abundance and diversity of the P-element during the invasion (gen.: generations) at at hot conditions in replicate 3. For details see caption of supplementary fig. 1.

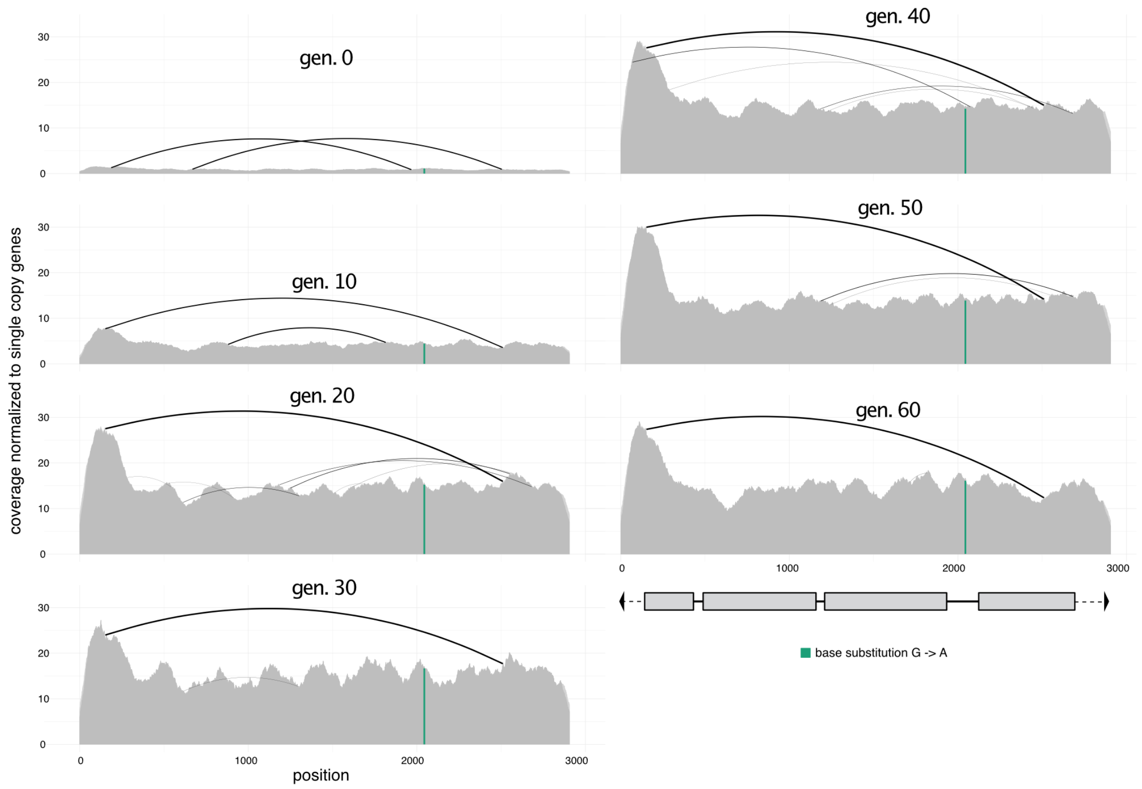

Figure 6: Abundance and diversity of the P-element during the invasion (gen.: generations) at hot conditions in replicate 5. For details see caption of supplementary fig. 1.

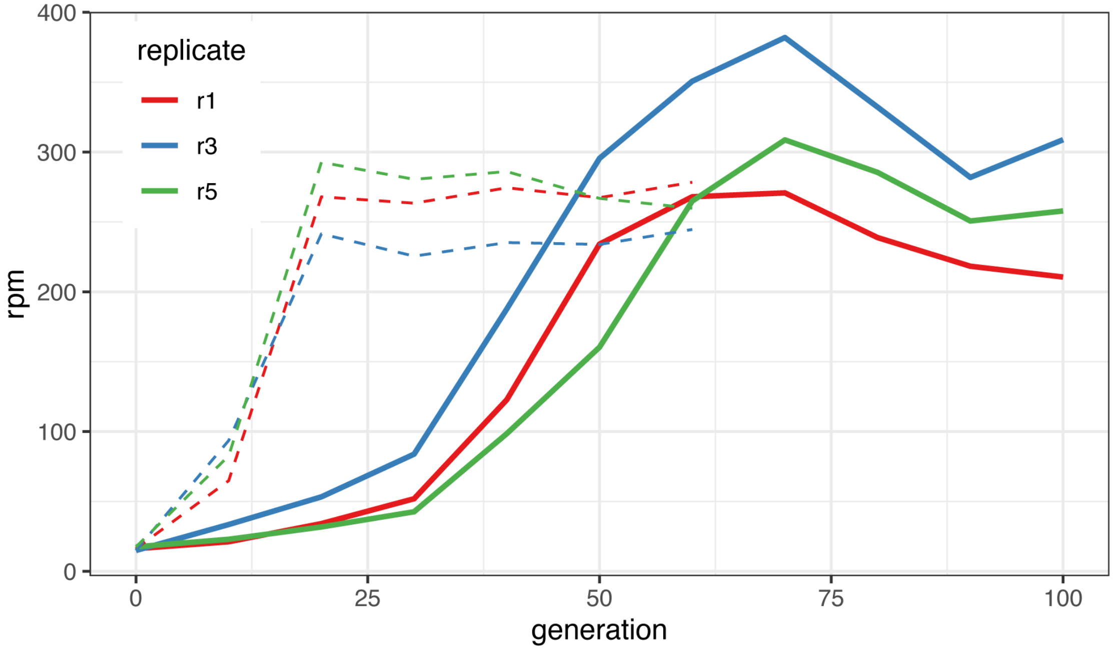

Figure 7: Abundance of P-element insertions during the invasion in three replicates. Here we provide raw estimates of the TE abundance in reads mapping to the P-element out of a million mapped reads (rpm). Hatched lines show the previously published invasion dynamics at hot conditions [Kofler et al., 2018].

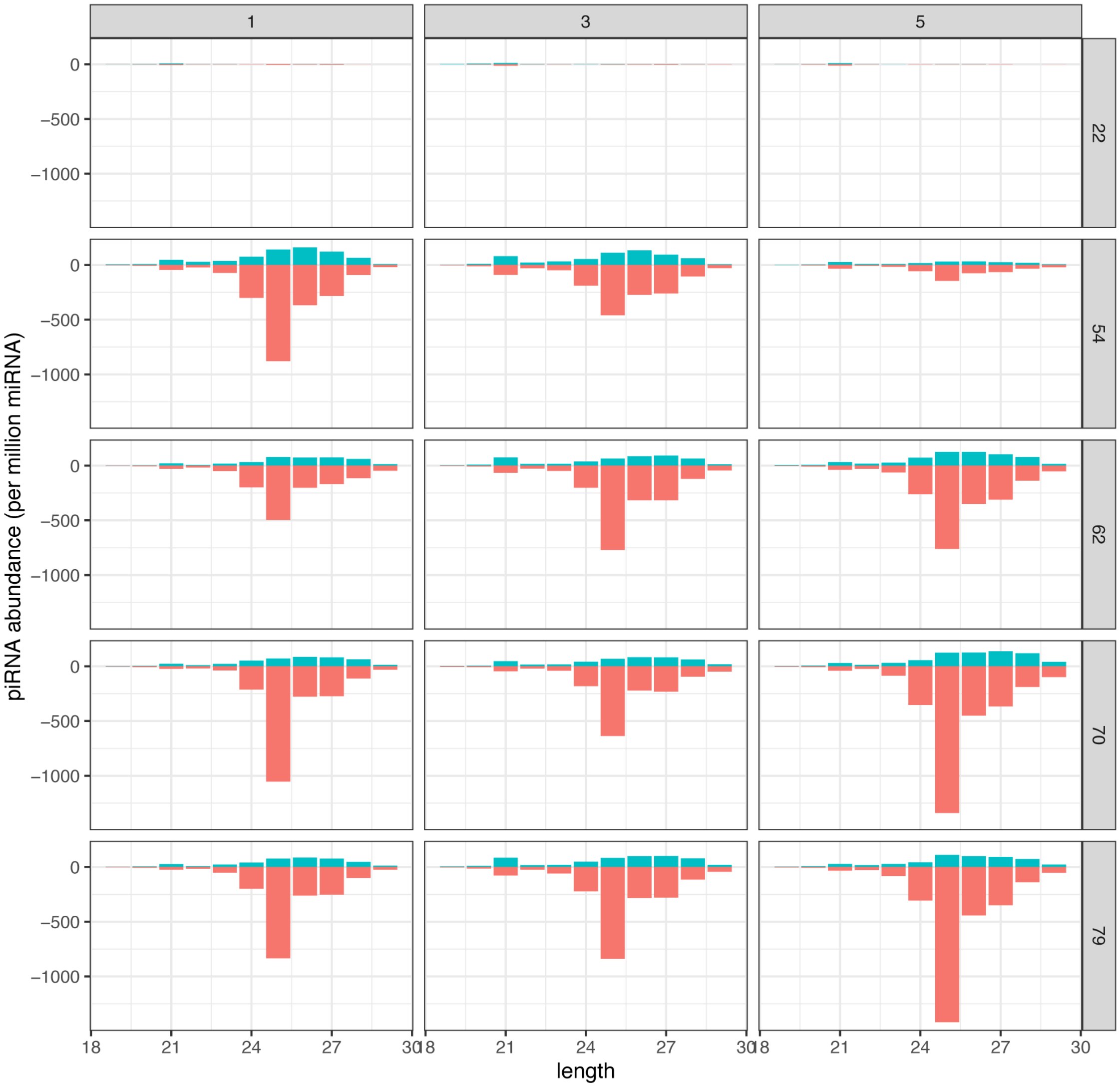

Figure 8: Length distribution of small RNAs (19-29nt) mapping to the P-element. The total abundance of small RNAs was normalized to 1 million miRNAs. Sense RNAs are on the positive y-axis (green) and antisense RNAs on the negative y-axis (red). The replicates are shown in the top panels and the generations in the right panels

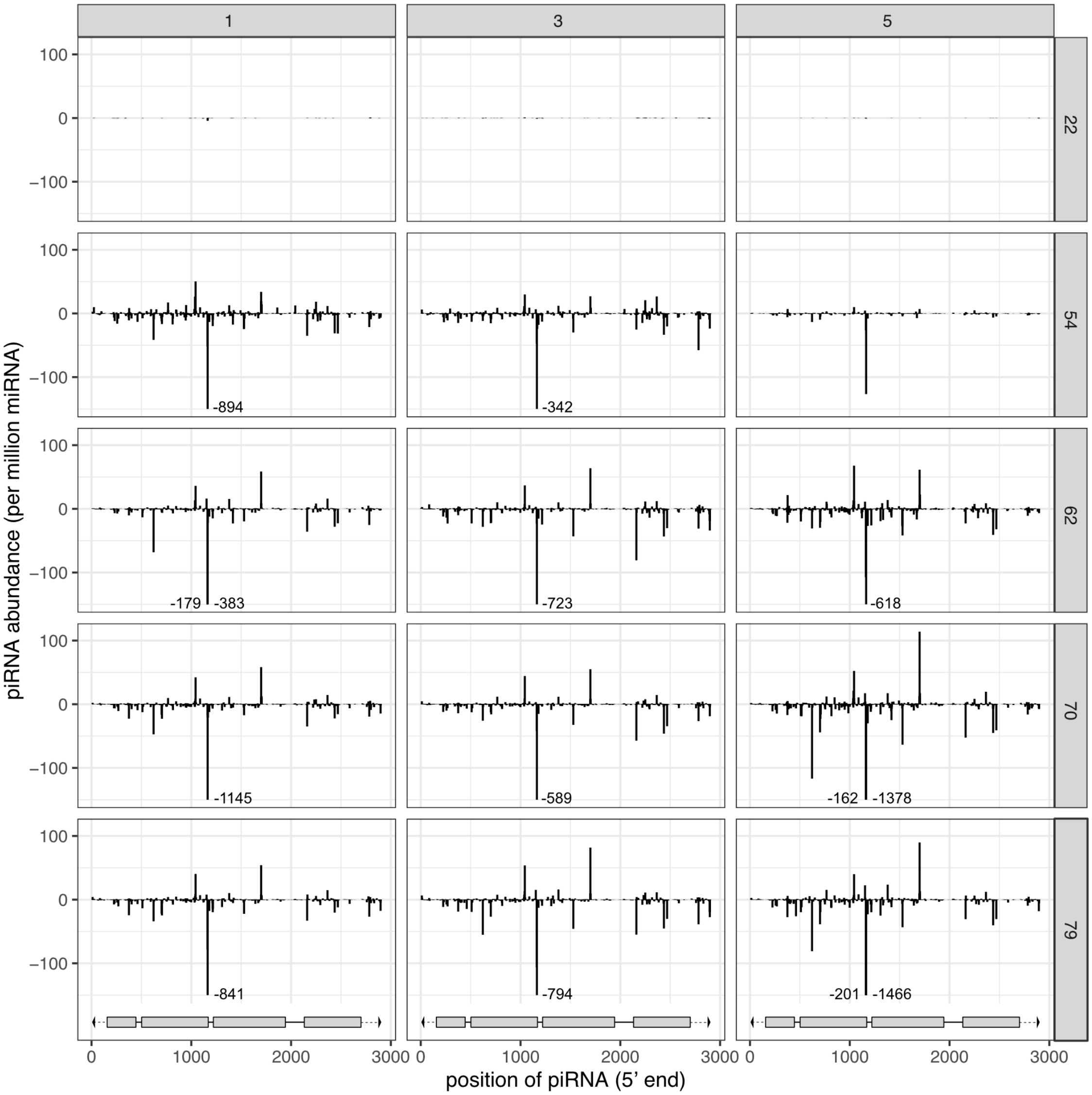

Figure 9: Distribution of piRNAs (23-29nt) along the P-element. Only the 5' positions of piRNAs are considered and piRNA abundance is normalized to one million miRNAs (ppm). Replicates are at the top panel and the generations at the right panel. Sense piRNAs are shown on the positive y-axis and antisense piRNAs on the negative y-axis. Large peaks were truncated (the total size of the peak is indicated). The structure of the P-element is indicated at the bottom, where we show TIRs (black triangles), the four ORFs (grey rectangles) and introns (black lines).

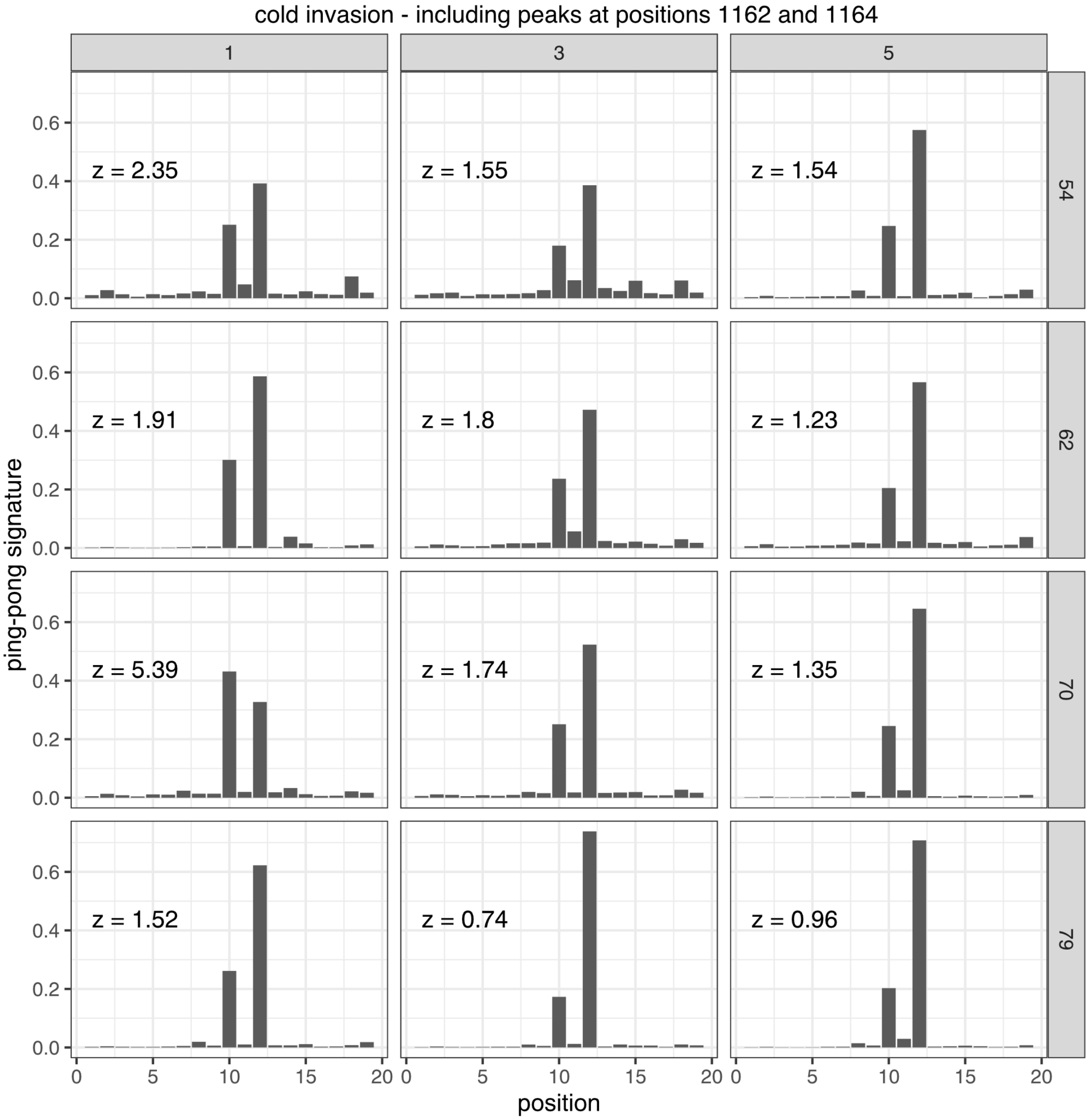

Figure 10: Ping-pong signature of P-element piRNAs during the cold invasion. The y-axis shows the fraction of pairs of sense and antisense piRNAs having the given overlap (x-axis) between the two 5' ends. Ping-pong z-scores are provided for each sample (e.g.: $z = 3.09$ corresponds to $p = 0.001$). Data are shown for the three replicates (top panel) at different generations during the experiment (right panel). Due to a low number of piRNAs we could not compute ping-pong signatures for any replicate at generation 22.

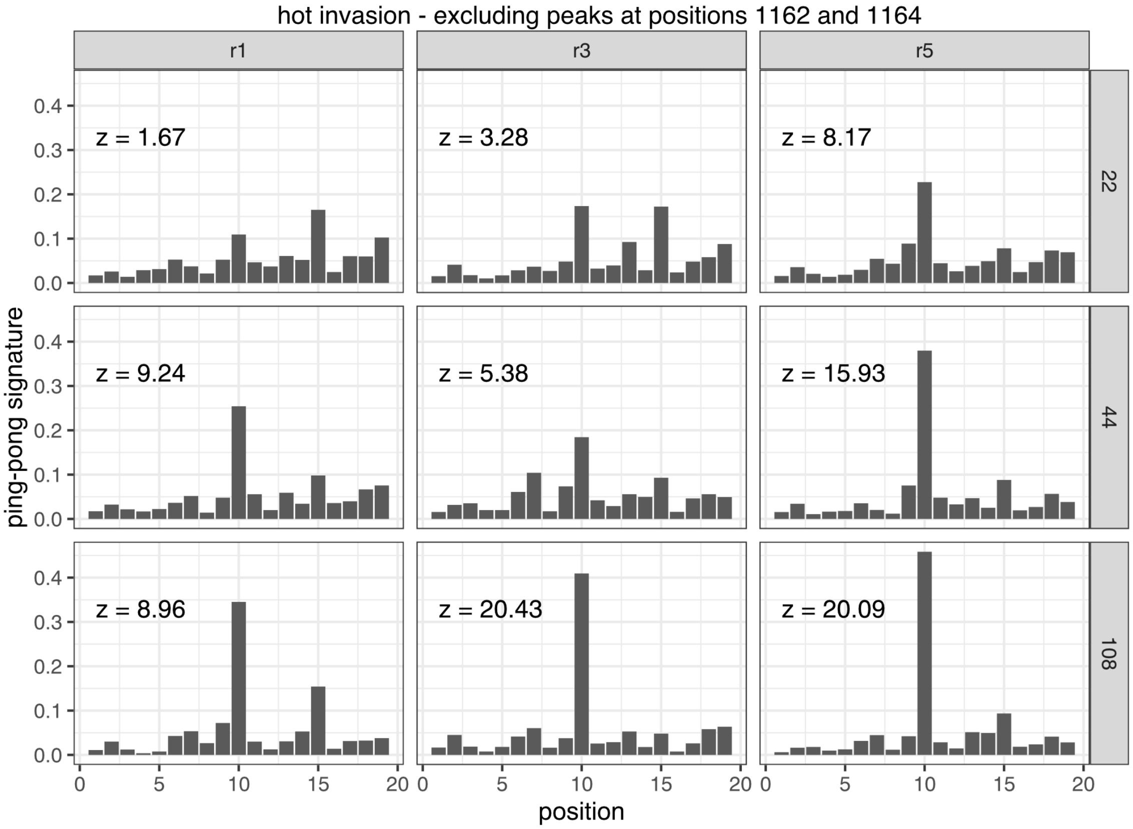

Figure 11: Ping-pong signature of P-element piRNAs during the hot invasion excluding piRNAs mapping to positions 1162 and 1164 (5'-end). The y-axis shows the fraction of pairs of sense and antisense piRNAs having the given overlap (x-axis) between the two 5' ends. Ping-pong z-scores are provided for each sample (e.g.: $z = 3.09$ corresponds to $p = 0.001$). Data are shown for the three replicates (top panel) at different generations during the experiment (right panel). Note that the ping-pong signature (at position 10) increases during the experiment.

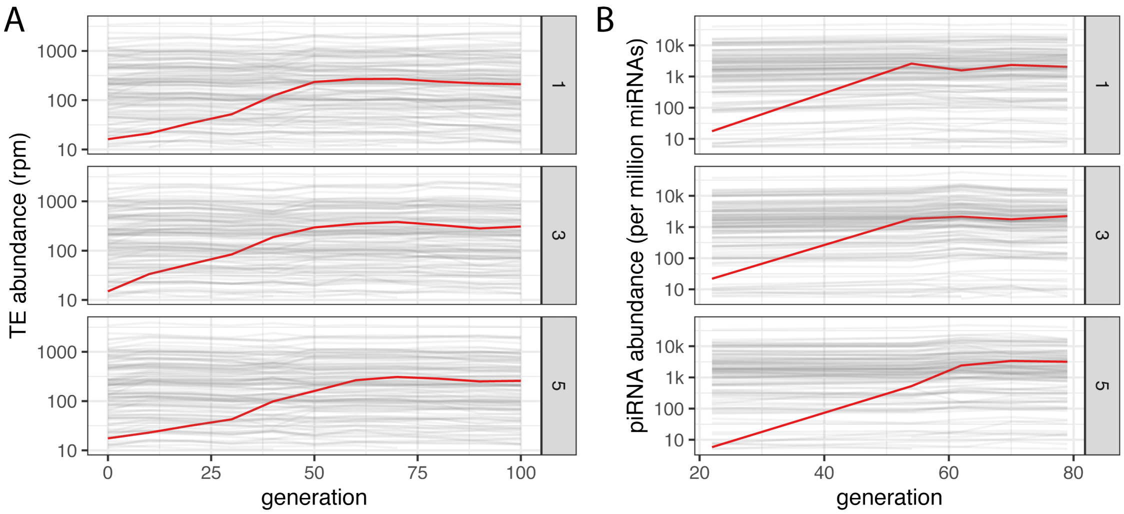

Figure 12: Abundance of TEs and piRNAs during the P-element (red) invasion at cold conditions. Data are shown for 121 TE families (grey; 23 TIR, 60 LTR, 37 non-LTR and 1 Foldback). A) Abundance of TEs in reads mapping to a TE per million reads (rpm). The base population consists solely of females whereas the evolved populations are a mix of males and females. Families with fewer than 10 rpm are not shown. B) Abundance of piRNAs in piRNAs per million miRNAs (ppm). Families with fewer than 5 ppm are not shown. Note that solely the P-element shows a marked increase in copy numbers and piRNAs.

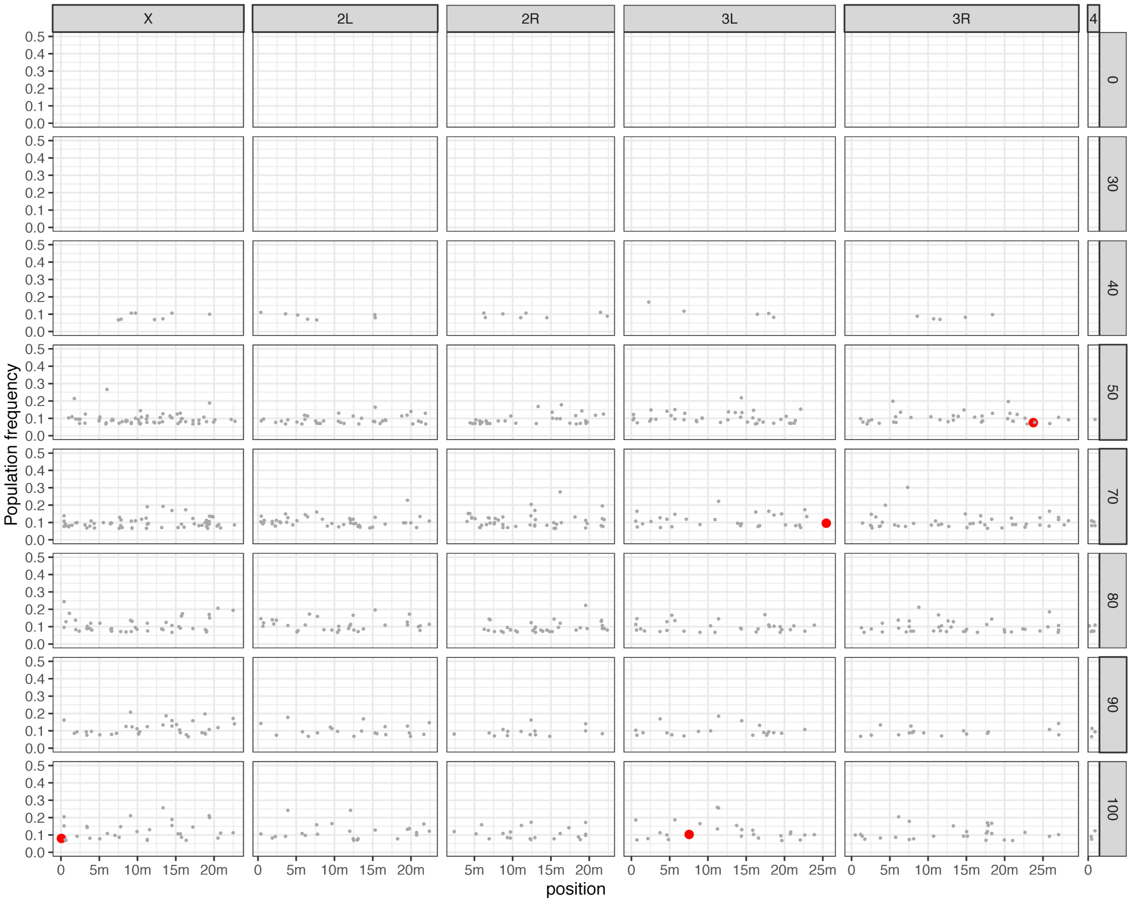

Figure 13: Positions and population frequencies of P-element insertions at cold conditions in replicate 1. The generations are in the right panel and P-element insertions in piRNA-clusters are shown red. Generations 10 and 20 are not shown due to the absence of detectable P-element insertions. To facilitate an unbiased comparison among samples we subsampled the physical coverage to 15. For generation 60 the physical coverage was insufficient. m: million base pairs

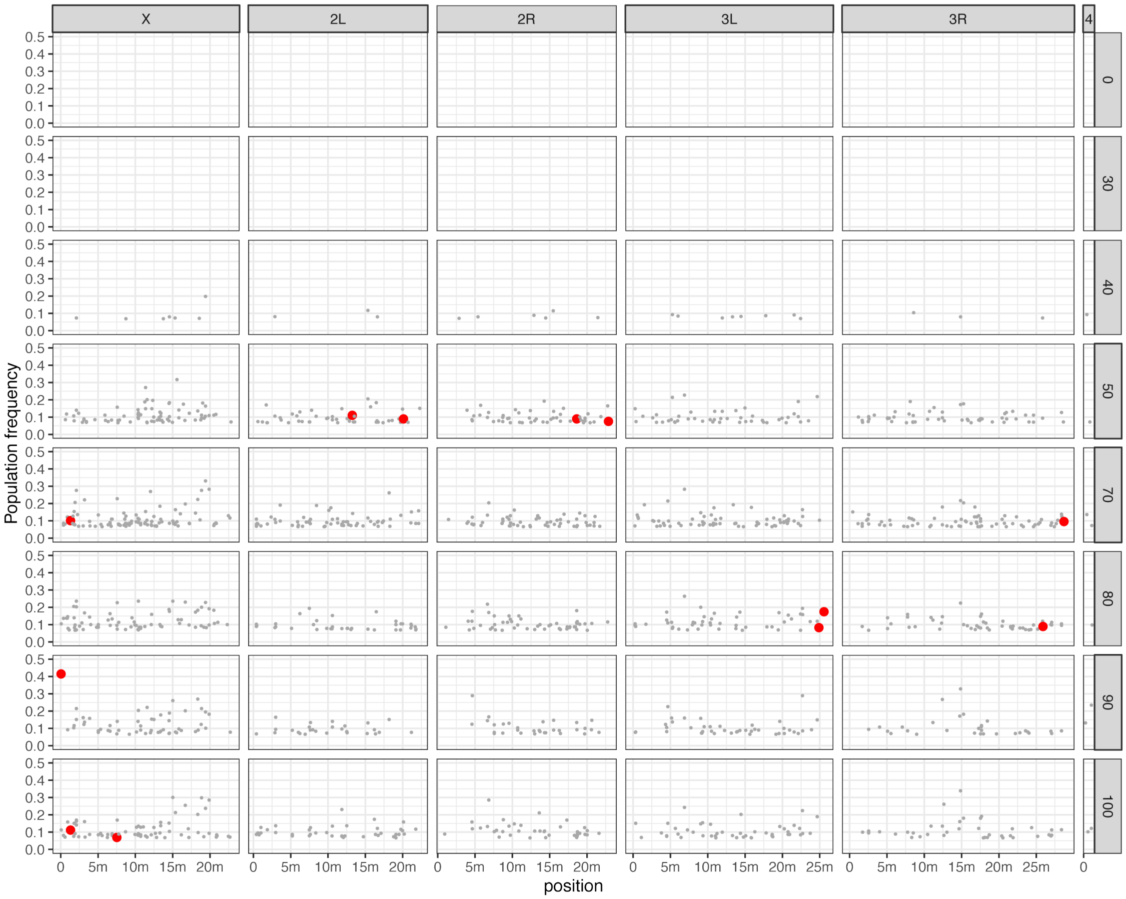

Figure 14: Positions and population frequencies of P-element insertions at cold conditions in replicate 3. For details see supplementary figure 13.

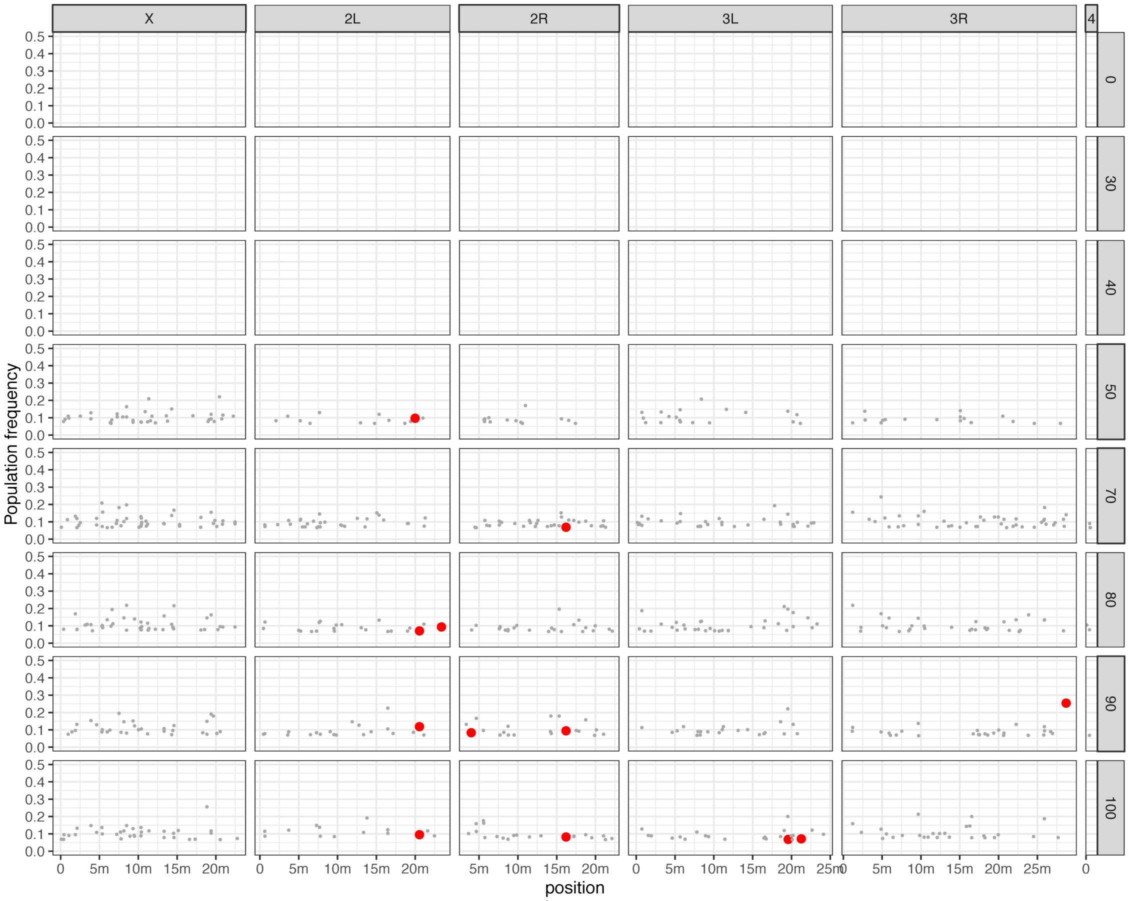

Figure 15: Positions and population frequencies of P-element insertions at cold conditions in replicate 5. For details see supplementary figure 13.

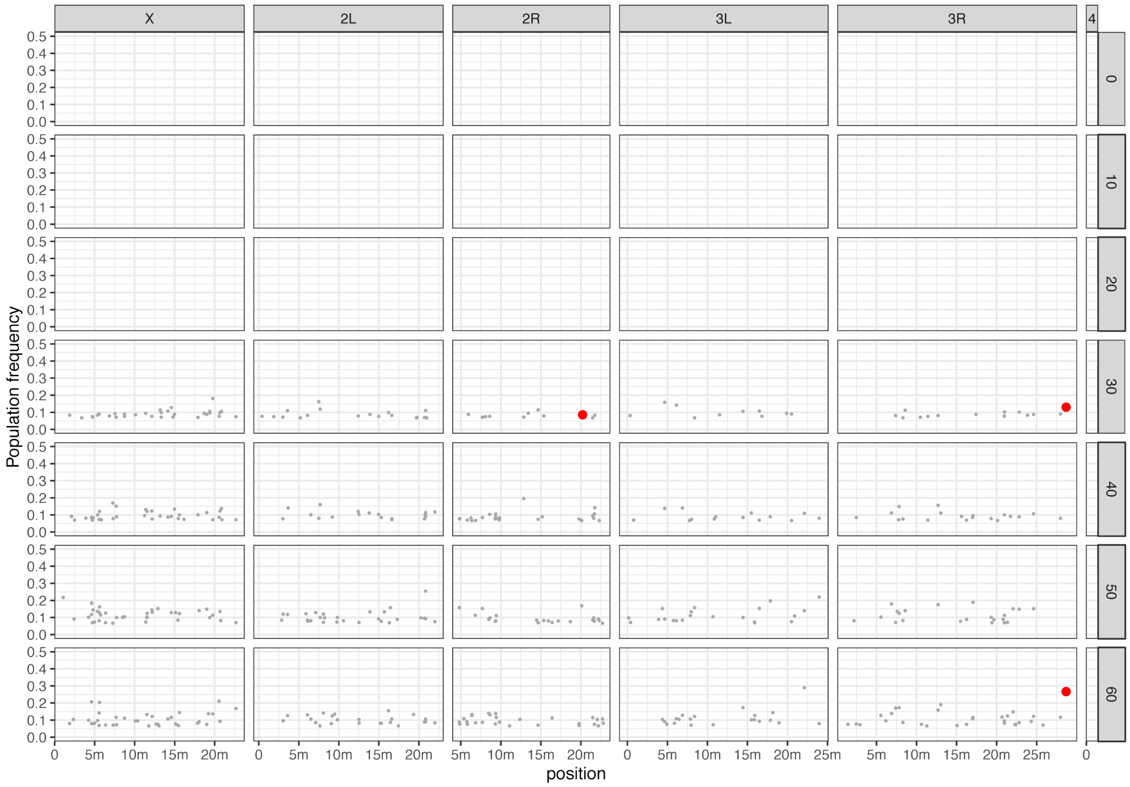

Figure 16: Positions and population frequencies of P-element insertions at hot conditions in replicate 1. The generations are in the right panel and P-element insertions in piRNA-clusters are shown red. We reanalyzed the data of our previous work [Kofler et al., 2018] with the slightly different pipeline used in this work (e.g. different read-length and reference genome) to facilitate a comparison of the P-element invasions at hot and cold conditions. The physical coverage was subsampled to 15. m: million base pairs

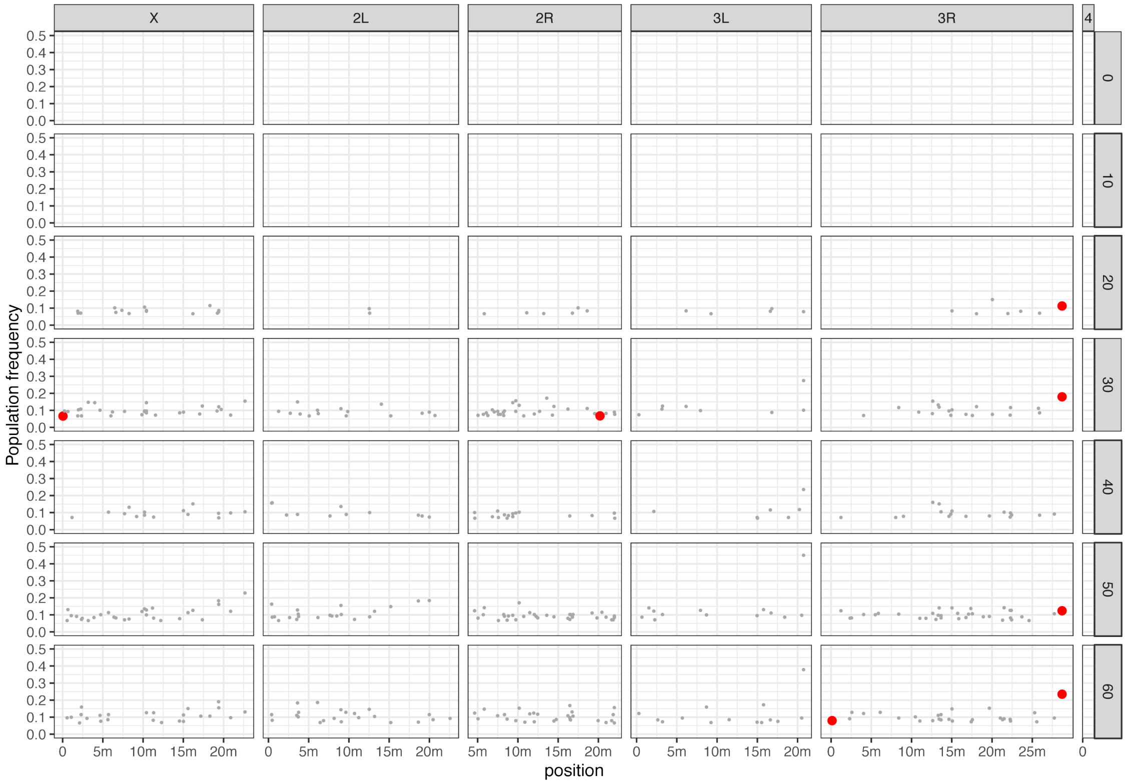

Figure 17: Positions and population frequencies of P-element insertions at hot conditions in replicate 3. For details see supplementary figure 16.

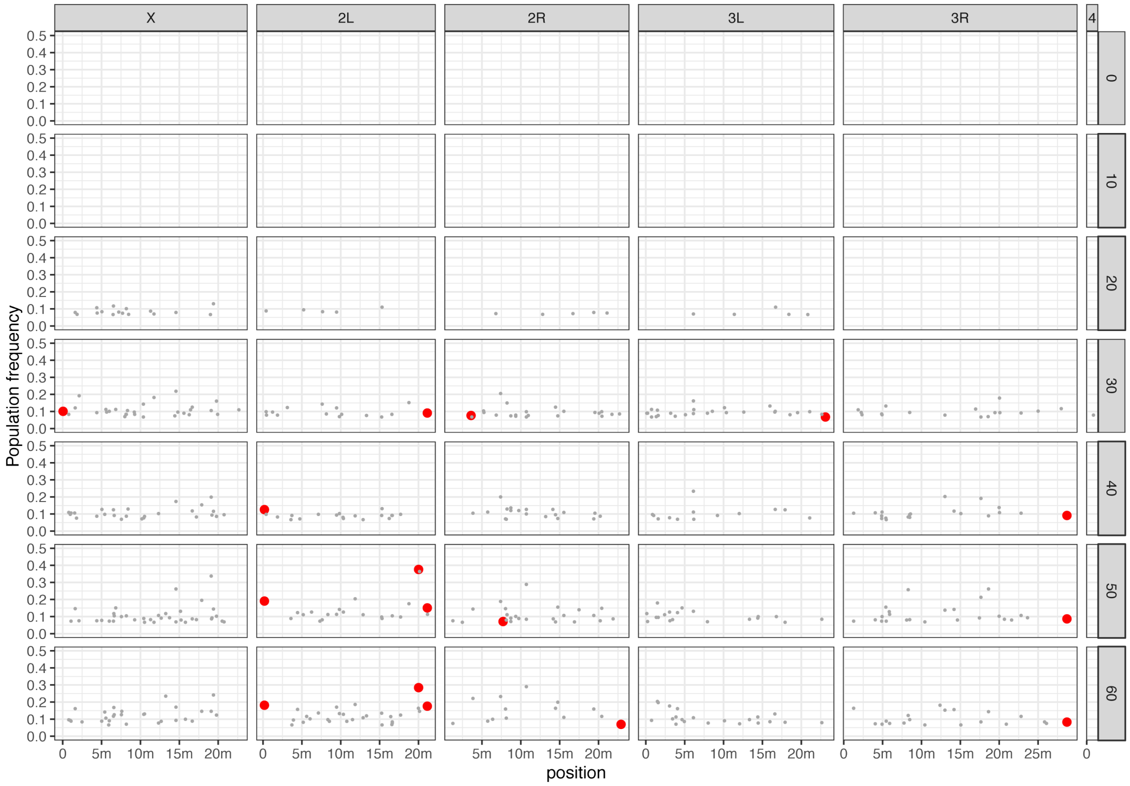

Figure 18: Positions and population frequencies of P-element insertions at hot conditions in replicate 5. For details see supplementary figure 16.

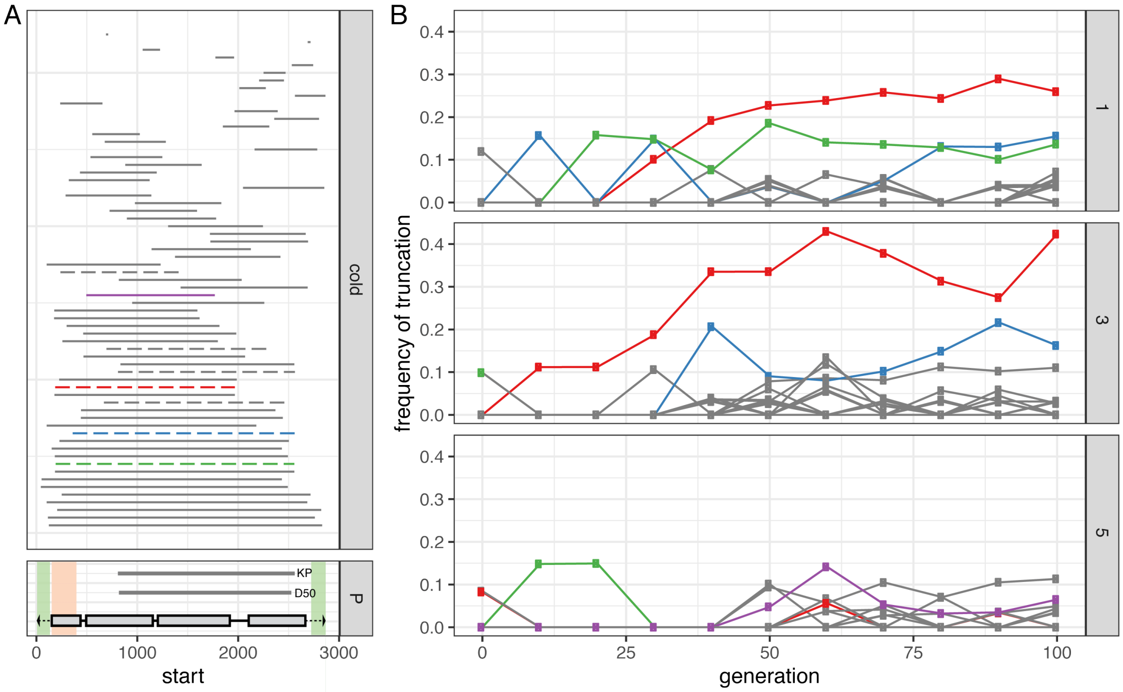

Figure 19: Dynamics of internally deleted P-elements during the invasion A) Positions of all internal deletions (IDs) observed during the cold invasion. Horizontal bars represents the deleted sequence. IDs that were likely present in the base population are shown as dashed line. The lower panel shows the structure of the P-element with the four ORFs (grey box) and the TIRs (black triangles). The position of two IDs that repress P-element activity (KP and D50) are indicated [Black et al., 1987, Rasmusson et al., 1993]. Regions required for mobilization of the P-element are shaded in green, and regions required for repressing P-element activity are shaded in orange [Majumdar and Rio, 2015]. B) Frequency of IDs during the invasion for all three replicates (right panel). The color of the IDs is identical to panel A.

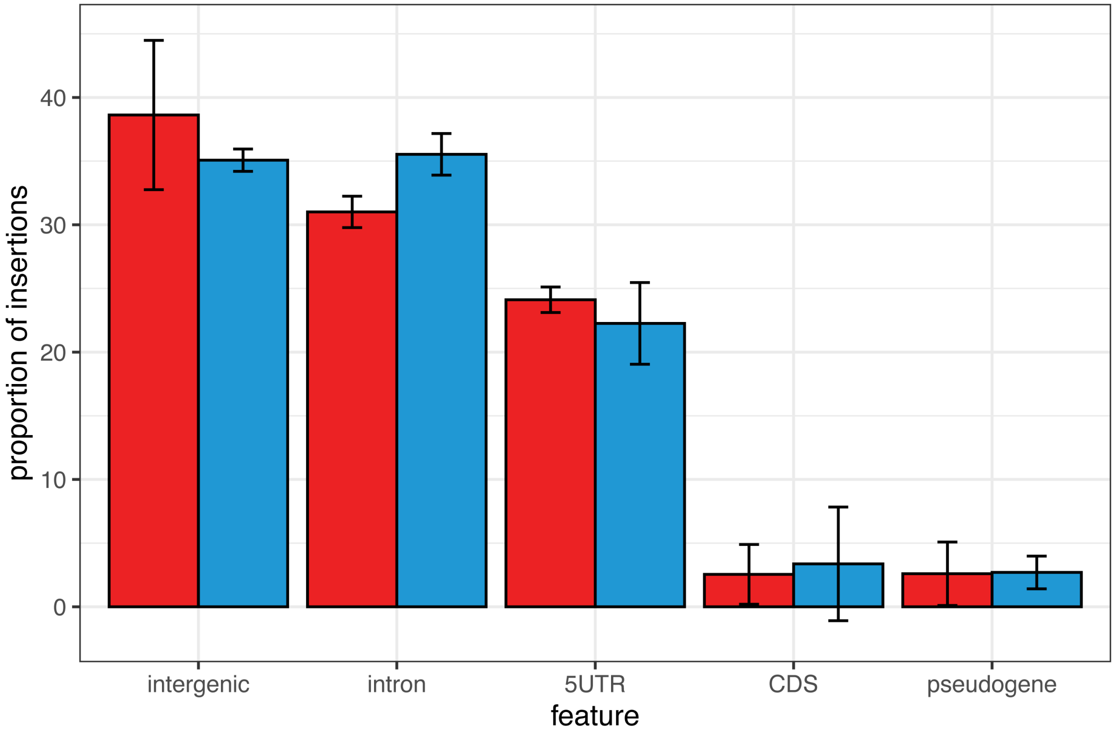

Figure 20: Proportion of P-element insertions in different genomic features at the end of the experimental invasions (hot invasion at generation 60, cold invasion at generation 100). Due to a low number of insertions results are not shown for 3'-UTR ($< 2\%$). The difference in the number of insertions in diverse genomic features between hot and cold conditions is not significant (Cochran-Mantel-Haenszel test based on the number of P-element insertions in each feature and replicate at two temperatures $df = 4$, $p = 0.62$).

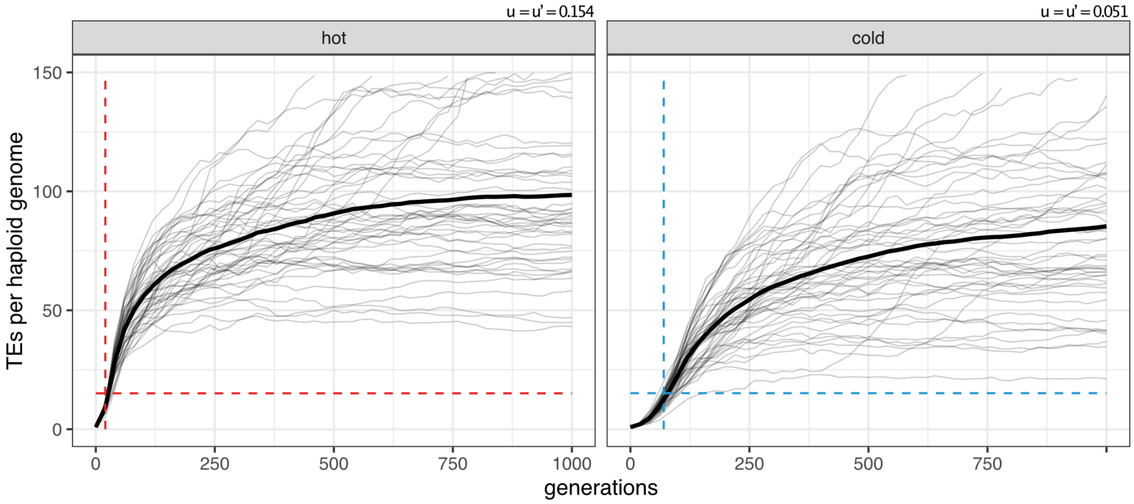

Figure 21: Expected and observed invasion dynamics under the trap model. The solid lines show the expected invasion dynamics with the parameters inferred from the data. Fifty replicates where used and the bold lines are averages. The dashed lines indicate the observed onset and level of the plateau in our experimental populations. Since neutral TE insertions were simulated ($x = 0$), the transposition rate (u) is identical to the effective transposition rate ($u' = u - x$).

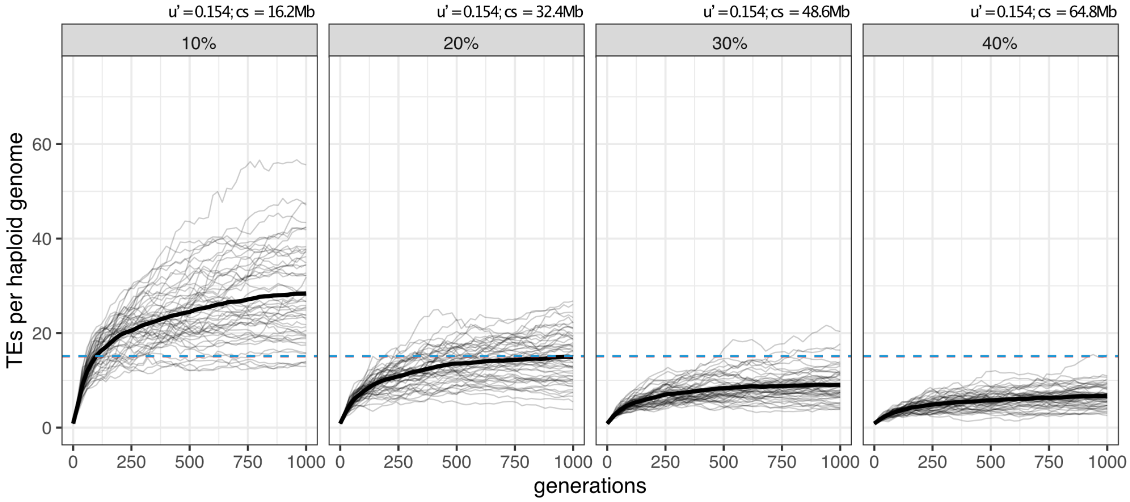

Figure 22: Influence of the size of piRNA clusters on the invasion dynamics. The solid lines show the expected invasion dynamics under the trap model. Fifty replicates where used and the bold lines are the average. The dashed lines indicate the plateau level in our experimental populations. Since neutral TE insertions were simulated, the transposition rate is identical to the effective transposition rate ($u' = u$). u' effective transposition rate, cs cluster size.

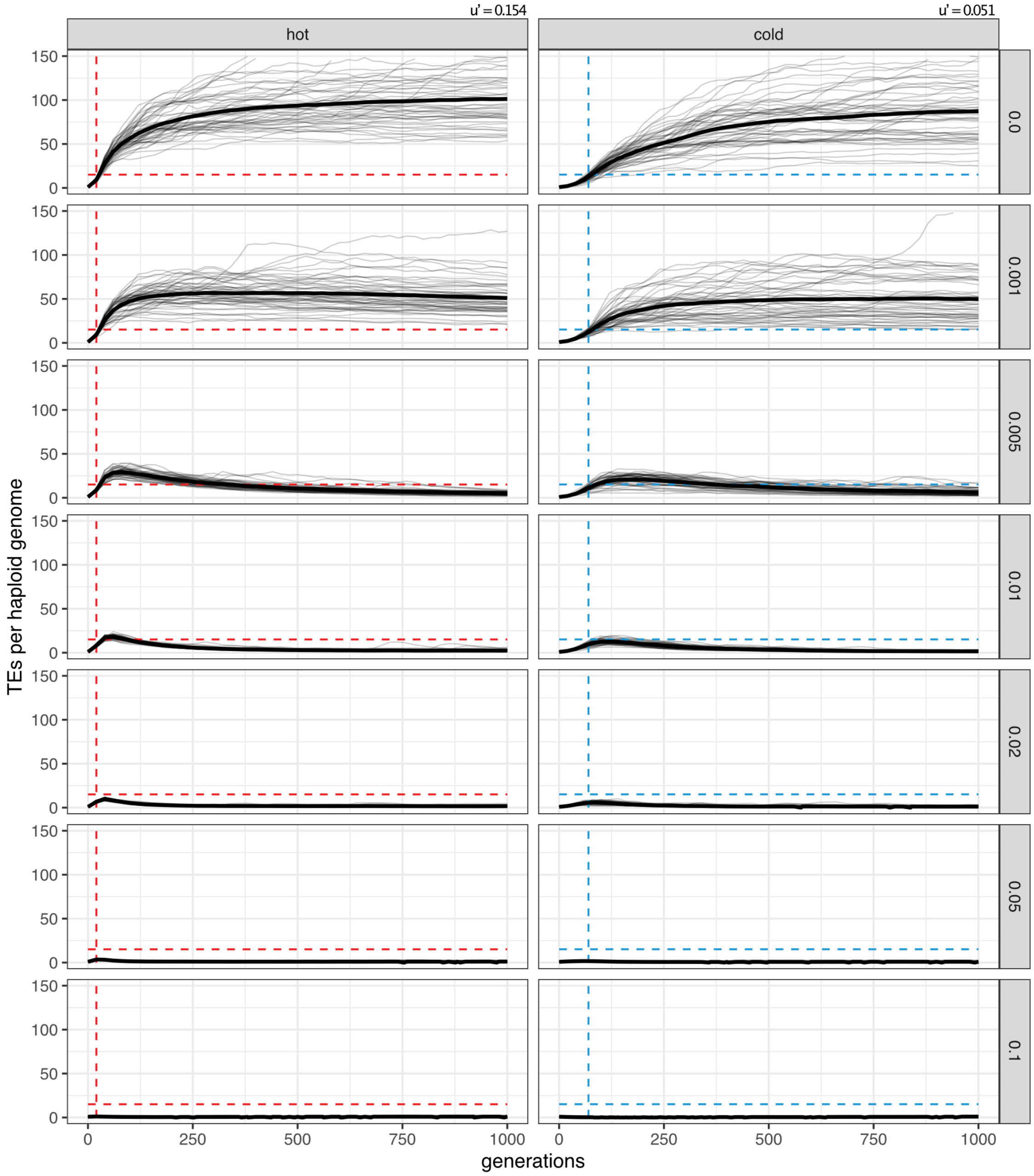

Figure 23: Expected and observed invasion dynamics under the trap model with constant negative effects of TE insertions (insertions in piRNA clusters were neutral). The effective transposition rate ($u' = u - x$) was kept constant by simultaneously changing the negative effect of TE insertions (x; right panel) and the transposition rate (u) . The solid lines show the expected invasion dynamics. Fifty replicates where used and the bold lines are averages. The dashed lines indicate the observed onset and level of the plateau in our experimental populations. u' effective transposition rate

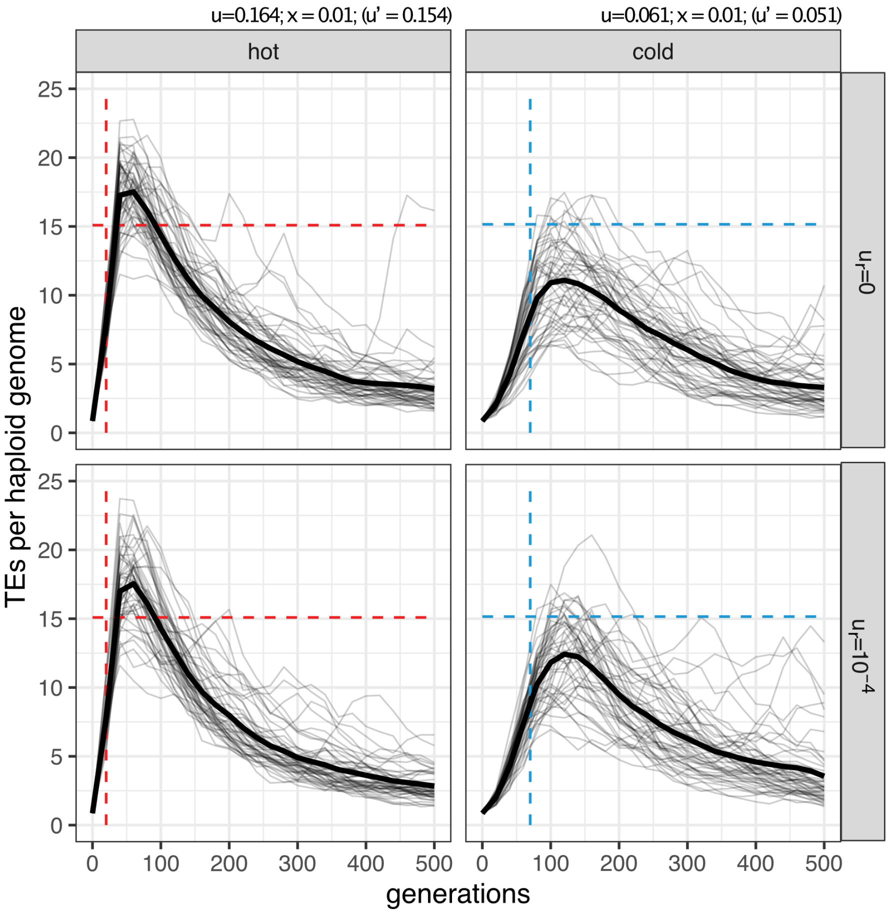

Figure 24: Influence of the residual activity. Results for 50 simulations are shown. The dashed lines indicate the observed onset and level of the plateau in our experimental populations. No significant influence of the residual activity was detected in hot and cold conditions (six Wilcoxon rank sum tests based on the TE abundance in simulations with and without residual activity at generations 60, 100 and 500; each $p > 0.10$). u transposition rate in individuals without a cluster insertion, u_r residual activity, i.e. transposition rate in individuals with a cluster insertion, x negative fitness effect of TEs. u' effective transposition rate, cs cluster size.

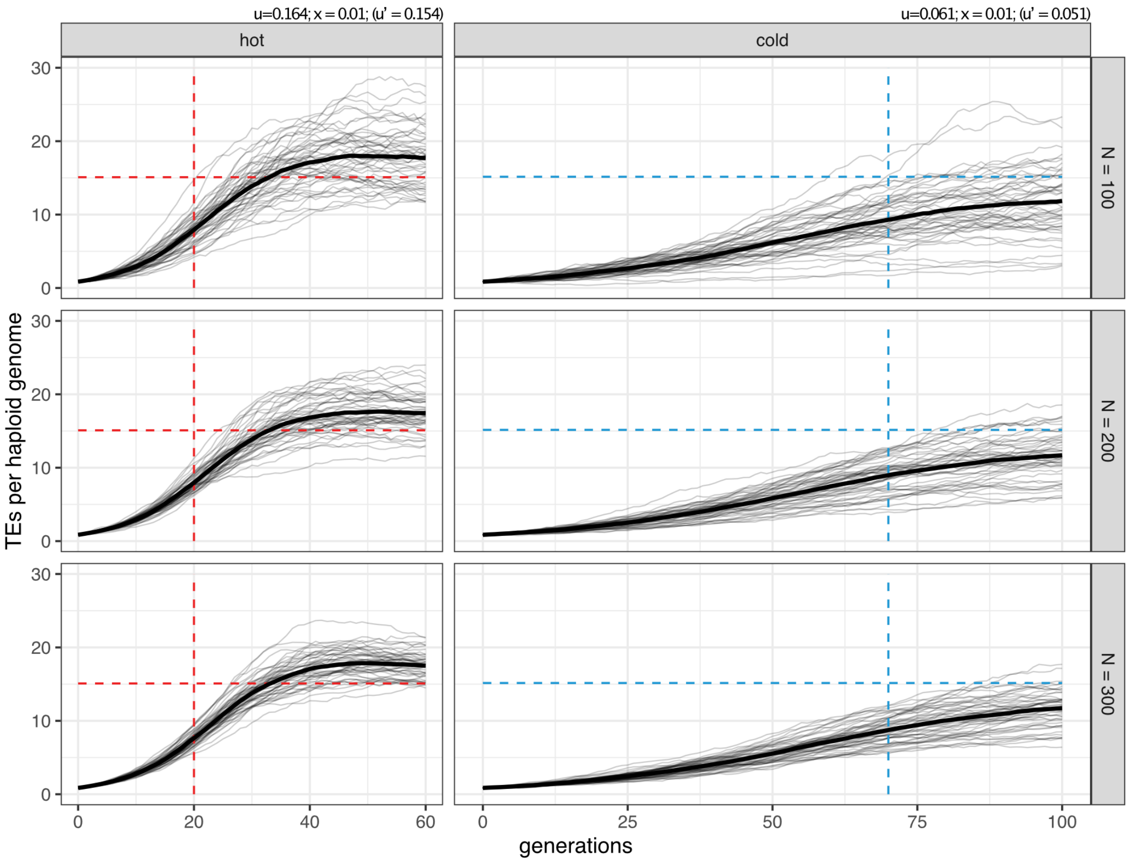

Figure 25: Influence of the effective population size (N; right panel). Results for 50 simulations are shown. The dashed lines indicate the observed onset and level of the plateau in our experimental populations.

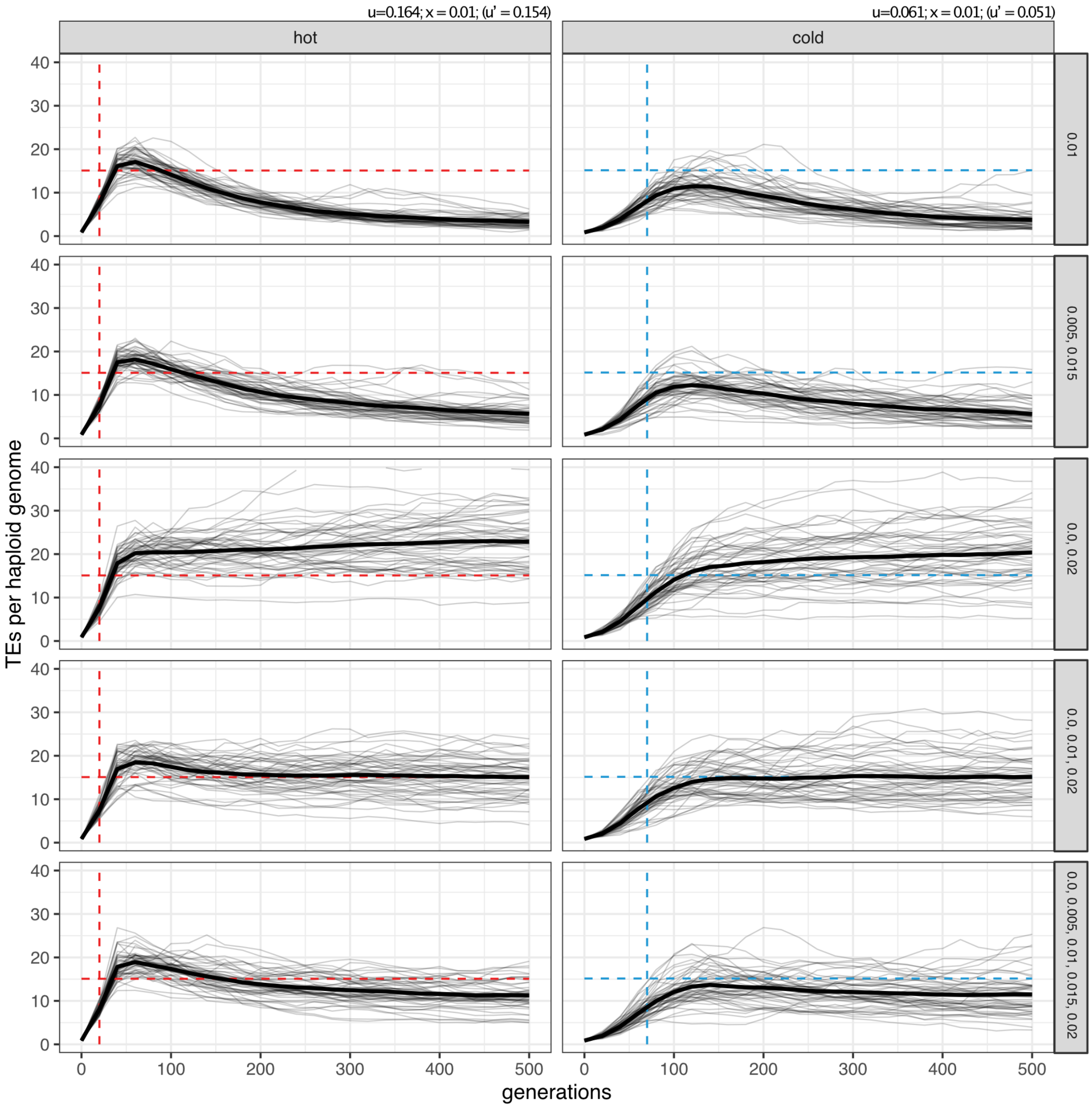

Figure 26: Expected and observed invasion dynamics under the trap model with a distribution of negative effects of TE insertions (the average negative effect was $\bar{x} = 0.01$; insertions in piRNA clusters were neutral). TE insertions had equal proportions of the negative effects indicated in the right panel. For example with "0.0, 0.01, 0.02", about 33.3% of the insertions had a negative effect of $x = 0.0$, another 33.3% had $x = 0.01$, and 33% had $x = 0.02$. The solid lines show the expected invasion dynamics under the trap model. Fifty replicates where used and the bold lines are averages. The dashed lines indicate the observed onset and level of the plateau in our experimental populations. u' effective transposition rate, cs cluster size.

Table 1: Overview of the Pool-Seq data used for estimating the status of the P-element invasion. We show the IDs of the lanes, the read length (rl.), the inner distance (id.) when reads are trimmed to length 75bp as used for finding the insertion sites with PoPoolationTE2 [Kofler et al., 2016], the number of sequenced paired end reads (in millions) for each replicate and the source publication of the data. Reads of several samples (e.g. 313a) were trimmed to a length of 100bp.

				.	replicate			
sample	lane IDs	Sequencer	rl.	id.	1	3	5	source
base	126a,b,c,d,e	HiSeq 2000	100	237	73.5	105.8	122.1	Kofler et al. [2018]
hot10	127a,b	HiSeq 2000	100	241	34.1	27.0	44.7	Kofler et al. [2018]
hot20	128a,b,c,d	HiSeq 2000	100	183	88.0	85.0	79.0	Kofler et al. [2018]
hot30	129b,c,d	HiSeq 2000	100	209	40.8	36.5	22.2	Kofler et al. [2018]
hot40	130b,c,d	HiSeq 2000	100	186	59.0	75.9	50.4	Kofler et al. [2018]
hot50	131b,c	HiSeq 2000	100	181	29.3	30.0	36.5	Kofler et al. [2018]
hot60	140b,c	HiSeq 2000	100	236	48.6	27.9	31.6	Kofler et al. [2018]
cold10	132b,c	HiSeq 2000	100	197	34.7	40.6	34.2	Kofler et al. [2018]
cold20	133b,c	HiSeq 2000	100	196	42.7	25.0	24.4	Kofler et al. [2018]
cold30	134b,c	HiSeq 2000	100	183	29.2	32.7	33.1	Kofler et al. [2018]
cold40	185a,b,c	HiSeq 2500	120(100)	360	19.4	46.9	21.7	Kofler et al. [2018]
cold50	313a	HiSeq X Ten	150(100)	131	38.7	41.0	38.8	this work
cold60	329a	HiSeq X Ten	150(100)	114	13.3	12.2	33.0	this work
cold70	393a	HiSeq X Ten	150(100)	108	39.9	45.3	50.9	this work
cold80	487a	HiSeq X Ten	150(100)	128	34.7	37.4	42.5	this work
cold90	542a	HiSeq X Ten	150(100)	173	40.0	42.0	40.0	this work
cold100	543a	HiSeq X Ten	150(100)	174	41.0	39.8	37.9	this work

Supplementary Tables

Table 2: Abundance and effective transposition rate (u') of the P-element in experimental evolving populations. Data are shown for different temperatures (temp), generations (gen) and replicates (columns 1, 3, 5). The abundance of the P-elements was estimated as reads per million (rpm) using PoPoolationTE2 [Kofler et al., 2016] and as insertions per haploid genome (ins) using DeviaTE [Weilguny and Kofler, 2019].

		rpm			ins			u'_{rpm}			u'_{ins}		
temp	gen	1	3	5	1	3	5	1	3	5	1	3	5
base	0	16.2	14.9	17.5	0.85	0.80	0.92	-	-	-	-	-	-
hot	10	64.9	93.6	82.6	3.89	5.32	4.38	0.149	0.201	0.168	0.164	0.209	0.169
hot	20	268.0	241.5	292.7	15.73	13.68	15.59	0.152	0.099	0.135	0.150	0.099	0.135
hot	30	263.4	225.5	280.5	15.94	13.08	16.20	-0.002	-0.007	-0.004	0.001	-0.004	0.004
hot	40	274.4	235.2	286.1	15.69	13.09	16.24	0.004	0.004	0.002	-0.002	0.000	0.000
hot	50	267.4	233.9	266.9	15.74	13.32	15.04	-0.003	-0.001	-0.007	0.000	0.002	-0.008
hot	60	278.4	244.6	259.8	17.35	14.04	15.64	0.004	0.004	-0.003	0.010	0.005	0.004
cold	10	21.2	33.5	22.9	1.21	1.83	1.20	0.027	0.084	0.027	0.036	0.086	0.027
cold	20	34.1	53.3	31.8	1.87	2.88	1.81	0.049	0.048	0.033	0.045	0.047	0.042
cold	30	51.9	83.9	42.6	3.17	5.11	2.41	0.043	0.046	0.030	0.054	0.059	0.029
cold	40	122.8	187.7	98.5	7.89	12.80	5.77	0.090	0.084	0.087	0.096	0.096	0.091
cold	50	234.1	295.5	160.4	12.80	16.78	8.54	0.067	0.046	0.050	0.050	0.027	0.040
cold	60	268.0	350.7	264.9	15.16	22.46	13.55	0.014	0.017	0.051	0.017	0.030	0.047
cold	70	270.8	381.9	308.7	15.25	21.65	15.49	0.001	0.009	0.015	0.001	-0.004	0.014
cold	80	238.8	332.1	285.4	12.77	18.08	13.63	-0.012	-0.014	-0.008	-0.018	-0.018	-0.013
cold	90	218.3	281.8	250.7	11.93	16.24	12.62	-0.009	-0.016	-0.013	-0.007	-0.011	-0.008
cold	100	210.6	308.8	257.9	12.14	18.16	13.87	-0.004	0.009	0.003	0.002	0.011	0.009

Table 3: Overview of small RNA data used in this work. We sequenced small RNA from whole flies at multiple time points during the experimental invasions and assessed the abundance of reads mapping to TEs (i.e. siRNAs and piRNAs), miRNAs, tRNAs, rRNAs and mRNAs. For each class of small RNAs we also estimated the fraction of reads mapping to the sense strand. Samples marked with a star (*) were published in a previous work [Kofler et al., 2018]. temp.: temperature, r.: replicate, gen.: generation, t.[M]: total number of reads in million, m.[M]: mapped reads in million

					abundance [%]					fraction sense[%]				
temp.	r.	gen.	t.[M]	m.[M]	TE	miRNA	tRNA	rRNA	mRNA	TE	miRNA	tRNA	rRNA	mRNA
hot*	1	22	14.0	7.5	22.4	58.8	8.4	1.4	9.0	25.6	100.0	100.0	99.8	61.0
hot*	3	22	13.7	6.1	18.4	63.1	7.2	2.7	8.5	26.8	100.0	100.0	99.9	67.6
hot*	5	22	14.6	7.6	22.2	65.7	2.7	2.3	7.1	26.4	100.0	100.0	99.8	51.4
hot*	1	44	13.8	6.5	15.8	40.4	21.5	9.5	12.9	23.9	100.0	100.0	100.0	81.5
hot*	3	44	14.2	7.7	15.8	40.0	24.5	7.8	11.9	25.5	100.0	100.0	100.0	79.2
hot*	5	44	13.6	7.1	24.6	49.7	6.9	10.6	8.2	23.6	100.0	100.0	100.0	58.4
hot*	1	108	12.8	6.8	27.2	60.5	1.1	4.4	6.9	25.2	100.0	99.9	99.9	35.0
hot*	3	108	14.7	7.2	18.0	70.2	1.6	5.7	4.6	25.1	100.0	99.9	100.0	42.1
hot*	5	108	15.2	7.6	25.9	60.0	1.1	6.5	6.5	25.0	100.0	99.8	100.0	37.3
cold*	1	22	12.7	5.0	17.4	51.0	11.8	9.0	10.8	24.9	100.0	100.0	100.0	76.0
cold*	3	22	18.5	7.5	16.1	50.0	8.4	10.6	14.9	23.5	100.0	100.0	100.0	83.3
cold*	5	22	17.7	7.3	18.7	56.3	5.8	12.0	7.2	24.5	100.0	100.0	100.0	64.3
cold*	1	54	12.3	6.1	25.5	61.7	1.2	5.8	5.9	24.6	100.0	99.9	99.9	40.5
cold*	3	54	14.4	7.8	23.7	61.9	1.8	6.6	6.0	24.8	100.0	99.9	100.0	42.6
cold*	5	54	16.7	8.5	21.8	62.8	1.8	8.2	5.3	23.1	100.0	99.9	100.0	38.2
cold	1	62	37.5	19.0	26.1	63.7	1.4	2.7	6.1	24.5	100.0	99.9	99.9	43.1
cold	3	62	46.4	24.1	29.0	59.8	0.9	3.4	7.0	24.7	100.0	99.8	99.9	37.1
cold	5	62	40.9	21.2	26.7	61.0	1.1	5.1	6.1	24.4	100.0	99.8	99.9	40.0
cold	1	70	35.0	18.2	26.5	61.7	1.7	4.3	5.9	24.6	100.0	99.9	99.9	43.5
cold	3	70	44.0	23.1	24.7	63.8	1.8	3.9	5.9	25.6	100.0	99.9	99.9	41.1
cold	5	70	43.1	22.1	26.2	63.2	1.5	3.0	6.1	25.0	100.0	99.9	99.9	40.0
cold	1	79	42.4	22.5	25.6	61.4	1.0	6.3	5.7	25.1	100.0	99.8	99.9	43.4
cold	3	79	39.8	19.9	24.0	64.6	1.2	4.3	5.8	25.8	100.0	99.9	99.9	40.7
cold	5	79	34.3	17.2	24.2	63.6	1.2	5.3	5.7	26.2	100.0	99.9	99.9	40.9

Table 4: Abundance of piRNAs (23-29nt) mapping to the P-element in the experimental populations. Data are shown for three replicates (r.) two temperatures (temp.) and multiple generations generations (gen.). The raw number of piRNAs mapping to the P-element (raw) and the number of piRNAs normalized to a million miRNA (norm) are shown.

temp.	r.	gen.	raw	norm
hot	1	22	8,377	2116.4
hot	1	44	5,949	2493.6
hot	1	108	13,131	3559.7
hot	3	22	7,230	2091.2
hot	3	44	5,522	1968.4
hot	3	108	12,317	2628.3
hot	5	22	9,353	2063.5
hot	5	44	12,649	3924.9
hot	5	108	16,695	4003.6
cold	1	22	41	17.7
cold	1	54	8,869	2593.1
cold	1	62	17,133	1568.5
cold	1	70	23,855	2340.4
cold	1	79	25,440	2046.5
cold	3	22	74	21.9
cold	3	54	8,056	1821.8
cold	3	62	27,707	2134.0
cold	3	70	23,395	1758.8
cold	3	79	25,865	2229.3
cold	5	22	22	5.8
cold	5	54	2,574	525.2
cold	5	62	28,091	2415.5
cold	5	70	42,549	3376.6
cold	5	79	31,186	3181.3

Table 5: Extent of gonadal dysgenesis during the P-element invasion. Results are shown at different generations for the three replicates. We estimated the number of flies having clearly visible ovarioles (normal), weakly visible ovarioles (intermediate), and no discernible ovarioles (dysgenic) at 29°C. Ovary dissections for each generation were done on a single day. The percentage of dysgenic ovaries is computed as $100 * (dysgenic + (intermediate/2))/(normal + intermediate + dysgenic)$

generation	replicate	normal	intermediate	dysgenic	dysgenic [%]
57	1	309	18	34	11.91
57	3	241	21	40	16.72
57	5	123	25	154	55.13
62	1	127	1	15	10.84
62	3	154	2	15	9.36
62	5	105	4	64	38.15
71	1	152	4	3	3.14
71	3	135	3	4	3.87
71	5	140	3	7	5.67
79	1	81	0	1	1.22
79	3	56	0	0	0.00
79	5	52	0	1	1.89

Table 6: Number of TE insertions in piRNA clusters and TAS regions; For each condition (cond), generation (g), replicate (r) we show an unbiased estimate of the number P-element insertions (Pi), the number of cluster insertions with and without TAS insertions (wTAS, woTAS) estimated by PoPoolationTE2, the number of TAS insertions based on anchor reads (anchor TAS; see supplementary table 8) and the sum of cluster and TAS insertions (cti $= woTAS + anchor\_TAS$).

				PopoolationTE2		anchor	
cond	g	r	Pi	wTAS	woTAS	TAS	cti
base	0	1	0.9	0.00	0.00	0.02	0.02
base	0	3	0.8	0.00	0.00	0.02	0.02
base	0	5	0.9	0.00	0.00	0.02	0.02
cold	10	1	1.2	0.00	0.00	0.05	0.05
cold	10	3	1.8	0.00	0.00	0.03	0.03
cold	10	5	1.2	0.00	0.00	0.08	0.08
cold	20	1	1.9	0.00	0.00	0.03	0.03
cold	20	3	2.9	0.00	0.00	0.05	0.05
cold	20	5	1.8	0.00	0.00	0.01	0.01
cold	30	1	3.2	0.00	0.00	0.03	0.03
cold	30	3	5.1	0.00	0.00	0.11	0.11
cold	30	5	2.4	0.00	0.00	0.13	0.13
cold	40	1	7.9	0.00	0.00	0.09	0.09
cold	40	3	12.8	0.00	0.00	0.24	0.24
cold	40	5	5.8	0.00	0.00	0.07	0.07
cold	50	1	12.8	0.08	0.08	0.16	0.23
cold	50	3	16.8	0.36	0.36	0.22	0.58
cold	50	5	8.5	0.10	0.10	0.13	0.22
cold	60	1	15.2	0.00	0.00	0.45	0.45
cold	60	3	22.5	0.00	0.00	0.27	0.27
cold	60	5	13.5	0.18	0.18	0.11	0.28
cold	70	1	15.2	0.10	0.10	0.32	0.42
cold	70	3	21.6	0.20	0.10	0.36	0.46
cold	70	5	15.5	0.07	0.07	0.23	0.30
cold	80	1	12.8	0.00	0.00	0.22	0.22
cold	80	3	18.1	0.35	0.35	0.22	0.57
cold	80	5	13.6	0.16	0.16	0.27	0.43
cold	90	1	11.9	0.00	0.00	0.17	0.17
cold	90	3	16.2	0.42	0.00	0.24	0.24
cold	90	5	12.6	0.55	0.30	0.15	0.44
cold	100	1	12.1	0.18	0.10	0.20	0.30
cold	100	3	18.2	0.18	0.18	0.19	0.37
cold	100	5	13.9	0.32	0.32	0.20	0.51
hot	10	1	3.9	0.00	0.00	0.08	0.08
hot	10	3	5.3	0.00	0.00	0.14	0.14
hot	10	5	4.4	0.00	0.00	0.16	0.16
hot	20	1	15.7	0.00	0.00	0.30	0.30
hot	20	3	13.7	0.11	0.00	0.59	0.59
hot	20	5	15.6	0.00	0.00	0.57	0.57
hot	30	1	15.9	0.22	0.09	0.45	0.53
hot	30	3	13.1	0.32	0.07	0.57	0.64
hot	30	5	16.2	0.34	0.24	0.50	0.74
hot	40	1	15.7	0.00	0.00	0.33	0.33
hot	40	3	13.1	0.00	0.00	0.63	0.63
hot	40	5	16.2	0.22	0.00	0.70	0.70
hot	50	1	15.7	0.00	0.00	0.32	0.32
hot	50	3	13.3	0.12	0.00	0.64	0.64
hot	50	5	15.0	0.88	0.60	0.64	1.24
hot	60	1	17.4	0.27	0.00	0.44	0.44
hot	60	3	14.0	0.31	0.08	0.56	0.64
hot	60	5	15.6	0.79	0.53	0.75	1.28

Table 7: Coordinates of TAS regions used in this work. We defined the region between the most distal gene and the end of the chromosome as TAS regions. chr. chromosome; spec. species from which the most distal gene (gene) was used, either *D. simulans* or *D. melanogaster*

name	chr.	start	end	length [bp]	gene	spec.
X-TAS	X	1	68,388	68,388	GD16489	Dsim
2L-TAS	2L	1	193,350	193,350	l(2)gl	Dmel
2R-TAS	2R	23,209,012	23,239,221	30,210	CG9380	Dmel
3L-TAS	3L	1	41,006	41,006	GD13543	Dsim
3R-TAS	3R	28,694,848	28,742,805	47,958	Map205	Dmel

Table 8: Overview of P-element insertions in TAS regions. For each condition (cond; hot/cold/base), generation (g.) and replicate we show the number of anchor reads mapping to X-TAS, 2L-TAS, 2R-TAS, 3L-TAS and 3R-TAS. Based on an unbiased estimate of the P-element abundance (Pi; using DeviaTE) and the fraction of anchor reads aligning to TAS regions the number of P-element insertions in TAS regions can be computed ($TAS\_insertions = Pi * TAS\_anchor\_reads/an$, where an is the total number of anchor reads). The enrichment of P-element insertions in TAS regions relative to random expectations was computed base on the total length of the genome and the length of the TAS regions.

					anchor reads in TAS						insertions in TAS						enrichment in TAS					
cond	g	r	Pi	an	all	X	2L	2R	3L	3R	all	X	2L	2R	3L	3R	all	X	2L	2R	3L	3R
base	0	1	0.85	251	7	5	1	0	0	1	0.02	0.02	0.00	0.00	0.00	0.00	10.7	42.8	3.0	0.0	0.0	12.2
base	0	3	0.80	336	8	4	3	0	0	1	0.02	0.01	0.01	0.00	0.00	0.00	9.2	25.5	6.8	0.0	0.0	9.1
base	0	5	0.92	428	11	6	2	0	0	3	0.02	0.01	0.00	0.00	0.00	0.01	9.9	30.1	3.5	0.0	0.0	21.5
cold	10	1	1.21	146	6	2	3	0	0	1	0.05	0.02	0.02	0.00	0.00	0.01	15.8	29.4	15.6	0.0	0.0	21.0
cold	10	3	1.83	230	4	3	1	0	0	0	0.03	0.02	0.01	0.00	0.00	0.00	6.7	28.0	3.3	0.0	0.0	0.0
cold	10	5	1.20	153	10	2	0	0	0	8	0.08	0.02	0.00	0.00	0.00	0.06	25.2	28.1	0.0	0.0	0.0	160.0
cold	20	1	1.87	294	4	1	2	0	0	1	0.03	0.01	0.01	0.00	0.00	0.01	5.2	7.3	5.2	0.0	0.0	10.4
cold	20	3	2.88	270	5	3	1	0	0	1	0.05	0.03	0.01	0.00	0.00	0.01	7.1	23.8	2.8	0.0	0.0	11.3
cold	20	5	1.81	160	1	1	0	0	0	0	0.01	0.01	0.00	0.00	0.00	0.00	2.4	13.4	0.0	0.0	0.0	0.0
cold	30	1	3.17	327	3	2	1	0	0	0	0.03	0.02	0.01	0.00	0.00	0.00	3.5	13.1	2.3	0.0	0.0	0.0
cold	30	3	5.11	538	12	8	4	0	0	0	0.11	0.08	0.04	0.00	0.00	0.00	8.6	31.9	5.6	0.0	0.0	0.0
cold	30	5	2.41	251	13	10	3	0	0	0	0.13	0.10	0.03	0.00	0.00	0.00	20.0	85.5	9.1	0.0	0.0	0.0
cold	40	1	7.89	806	9	9	0	0	0	0	0.09	0.09	0.00	0.00	0.00	0.00	4.3	24.0	0.0	0.0	0.0	0.0
cold	40	3	12.80	3023	56	31	17	1	0	7	0.24	0.13	0.07	0.00	0.00	0.03	7.1	22.0	4.3	1.6	0.0	7.1
cold	40	5	5.77	609	7	2	2	0	0	3	0.07	0.02	0.02	0.00	0.00	0.03	4.4	7.0	2.5	0.0	0.0	15.1
cold	50	1	12.80	2007	25	17	6	0	0	2	0.16	0.11	0.04	0.00	0.00	0.01	4.8	18.2	2.3	0.0	0.0	3.0
cold	50	3	16.78	2862	37	19	12	0	0	6	0.22	0.11	0.07	0.00	0.00	0.04	5.0	14.2	3.2	0.0	0.0	6.4
cold	50	5	8.54	1136	17	10	5	0	0	2	0.13	0.08	0.04	0.00	0.00	0.02	5.8	18.9	3.3	0.0	0.0	5.4
cold	60	1	15.16	914	27	15	7	0	0	5	0.45	0.25	0.12	0.00	0.00	0.08	11.4	35.2	5.8	0.0	0.0	16.7
cold	60	3	22.46	1594	19	6	7	0	0	6	0.27	0.08	0.10	0.00	0.00	0.08	4.6	8.1	3.3	0.0	0.0	11.5
cold	60	5	13.55	2437	19	8	8	0	0	3	0.11	0.04	0.04	0.00	0.00	0.02	3.0	7.0	2.5	0.0	0.0	3.8
cold	70	1	15.25	2091	44	22	18	0	0	4	0.32	0.16	0.13	0.00	0.00	0.03	8.1	22.6	6.5	0.0	0.0	5.9
cold	70	3	21.65	4469	75	44	23	0	0	8	0.36	0.21	0.11	0.00	0.00	0.04	6.5	21.1	3.9	0.0	0.0	5.5
cold	70	5	15.49	2700	40	18	7	0	0	15	0.23	0.10	0.04	0.00	0.00	0.09	5.7	14.3	2.0	0.0	0.0	17.0
cold	80	1	12.77	2015	35	15	19	0	0	1	0.22	0.10	0.12	0.00	0.00	0.01	6.7	16.0	7.2	0.0	0.0	1.5
cold	80	3	18.08	3066	38	30	7	0	0	1	0.22	0.18	0.04	0.00	0.00	0.01	4.8	21.0	1.7	0.0	0.0	1.0
cold	80	5	13.63	2223	44	17	11	0	0	16	0.27	0.10	0.07	0.00	0.00	0.10	7.6	16.4	3.8	0.0	0.0	22.0
cold	90	1	11.93	3672	53	20	17	0	0	16	0.17	0.06	0.06	0.00	0.00	0.05	5.6	11.7	3.5	0.0	0.0	13.3
cold	90	3	16.24	4416	64	47	8	0	0	9	0.24	0.17	0.03	0.00	0.00	0.03	5.6	22.8	1.4	0.0	0.0	6.2
cold	90	5	12.62	2465	29	11	5	0	0	13	0.15	0.06	0.03	0.00	0.00	0.07	4.5	9.6	1.5	0.0	0.0	16.1
cold	100	1	12.14	2929	48	27	4	0	0	17	0.20	0.11	0.02	0.00	0.00	0.07	6.3	19.8	1.0	0.0	0.0	17.8
cold	100	3	18.16	4400	46	33	10	0	0	3	0.19	0.14	0.04	0.00	0.00	0.01	4.0	16.1	1.7	0.0	0.0	2.1
cold	100	5	13.87	3037	43	13	17	0	0	13	0.20	0.06	0.08	0.00	0.00	0.06	5.5	9.2	4.2	0.0	0.0	13.1
hot	10	1	3.89	503	10	5	4	0	0	1	0.08	0.04	0.03	0.00	0.00	0.01	7.7	21.3	6.0	0.0	0.0	6.1
hot	10	3	5.32	514	14	11	2	0	0	1	0.14	0.11	0.02	0.00	0.00	0.01	10.5	45.9	3.0	0.0	0.0	6.0
hot	10	5	4.38	779	29	26	1	1	0	1	0.16	0.15	0.01	0.01	0.00	0.01	14.3	71.6	1.0	6.2	0.0	3.9
hot	20	1	15.73	4333	82	53	20	0	0	9	0.30	0.19	0.07	0.00	0.00	0.03	7.3	26.3	3.5	0.0	0.0	6.4
hot	20	3	13.68	3669	158	90	33	0	0	35	0.59	0.34	0.12	0.00	0.00	0.13	16.6	52.6	6.8	0.0	0.0	29.2
hot	20	5	15.59	4354	158	91	54	0	1	12	0.57	0.33	0.19	0.00	0.00	0.04	14.0	44.9	9.4	0.0	0.8	8.4
hot	30	1	15.94	2866	80	55	12	0	0	13	0.45	0.31	0.07	0.00	0.00	0.07	10.8	41.2	3.2	0.0	0.0	13.9
hot	30	3	13.08	2124	93	57	15	0	0	21	0.57	0.35	0.09	0.00	0.00	0.13	16.9	57.6	5.4	0.0	0.0	30.3
hot	30	5	16.20	1646	51	36	11	0	0	4	0.50	0.35	0.11	0.00	0.00	0.04	11.9	46.9	5.1	0.0	0.0	7.4
hot	40	1	15.69	3276	68	36	23	0	0	9	0.33	0.17	0.11	0.00	0.00	0.04	8.0	23.6	5.3	0.0	0.0	8.4
hot	40	3	13.09	3697	178	87	52	0	3	36	0.63	0.31	0.18	0.00	0.01	0.13	18.6	50.5	10.7	0.0	2.9	29.8
hot	40	5	16.24	2636	114	59	34	0	0	21	0.70	0.36	0.21	0.00	0.00	0.13	16.7	48.0	9.8	0.0	0.0	24.4
hot	50	1	15.74	1575	32	15	8	0	0	9	0.32	0.15	0.08	0.00	0.00	0.09	7.8	20.4	3.9	0.0	0.0	17.5
hot	50	3	13.32	1324	64	35	18	0	0	11	0.64	0.35	0.18	0.00	0.00	0.11	18.6	56.7	10.3	0.0	0.0	25.4
hot	50	5	15.04	1744	74	32	32	0	1	9	0.64	0.28	0.28	0.00	0.01	0.08	16.3	39.4	13.9	0.0	2.1	15.8
hot	60	1	17.35	3058	78	36	13	0	0	29	0.44	0.20	0.07	0.00	0.00	0.16	9.8	25.3	3.2	0.0	0.0	29.0
hot	60	3	14.04	1541	62	29	20	0	1	12	0.56	0.26	0.18	0.00	0.01	0.11	15.5	40.4	9.9	0.0	2.3	23.8
hot	60	5	15.64	1722	83	42	31	0	0	10	0.75	0.38	0.28	0.00	0.00	0.09	18.6	52.3	13.7	0.0	0.0	17.8

Table 9: Estimates of the effective population size (Ne) for the experimental populations. The estimates are not significantly different between hot and cold conditions (Welch Two Sample t-test $p = 0.74$).

replicate	hot	cold
1	297	229
3	179	264
5	188	216
mean	221	236

Supplementary results 1 - Liberal estimate of P-element insertions in piRNA clusters

Since we cannot rule out that cluster insertions in highly repetitive regions were missed, we estimated the number of cluster insertions using the most liberal criteria: we considered ambiguously mapped small RNAs for defining the positions of piRNA clusters and considered ambiguously mapped anchor reads within TAS as well as piRNA clusters. Even with these liberal criteria each individual carries only about 1.46 cluster/TAS insertions at cold conditions and 1.63 at hot conditions, still less than the two insertions required for reliable silencing of the P-element (supplementary table 10).

Although we used a long-read based assembly, some piRNA clusters may be missing in our assembly. Furthermore the position and composition of piRNA clusters is likely polymorphic [Wierzbicki et al., 2021a,b, Gebert et al., 2021] and therefore the sequences of piRNA clusters in our experimental populations may differ from the assembled ones. At cold (hot) conditions about 8.1% (11.2%) of the anchor reads can not be aligned to the reference genome (supplementary table 11). Assuming that equal proportions of aligned and not-aligned anchor reads map to clusters/TAS then each individual could, as an upper estimate, carry about 1.58 cluster/TAS insertions at cold and 1.81 at hot conditions (at the plateau). This liberal estimate of the number of cluster insertions takes into account i) TAS regions ii) ambiguous mapping of piRNAs and TE insertions and iii) polymorphism of piRNA clusters. The actual number of cluster insertions may only be higher than this estimate if a large fraction of the not-aligned anchor reads maps to piRNA clusters or we missed some piRNA clusters, e.g. strain specific clusters or lowly-expressed clusters.

Table 10: Upper estimate of the number of piRNA-producing P-element insertions (sum) in the experimental populations. Anchor reads were mapped to the sequences of TAS regions and piRNA clusters. We used both ambiguously and unambiguously mapped anchor reads. Also the annotation of the piRNA clusters was based on ambiguously and unambiguously aligned piRNAs.

				anchor			insertions		
	g	r	Pi	all	clu	tas	clu	tas	sum
base	0	1	0.85	251	11	6	0.04	0.02	0.06
base	0	3	0.80	336	22	8	0.05	0.02	0.07
base	0	5	0.92	427	31	7	0.07	0.02	0.08
cold	10	1	1.21	146	14	4	0.12	0.03	0.15
cold	10	3	1.83	230	16	2	0.13	0.02	0.14
cold	10	5	1.20	153	8	9	0.06	0.07	0.13
cold	20	1	1.87	295	14	4	0.09	0.03	0.11
cold	20	3	2.88	270	17	4	0.18	0.04	0.22
cold	20	5	1.81	160	12	1	0.14	0.01	0.15
cold	30	1	3.17	327	31	1	0.30	0.01	0.31
cold	30	3	5.11	538	35	7	0.33	0.07	0.40
cold	30	5	2.41	248	19	6	0.18	0.06	0.24
cold	40	1	7.89	806	35	5	0.34	0.05	0.39
cold	40	3	12.80	3023	193	31	0.82	0.13	0.95
cold	40	5	5.77	610	60	5	0.57	0.05	0.61
cold	50	1	12.80	2008	117	21	0.75	0.13	0.88
cold	50	3	16.78	2867	182	26	1.07	0.15	1.22
cold	50	5	8.54	1136	90	9	0.68	0.07	0.74
cold	60	1	15.16	914	88	21	1.46	0.35	1.81
cold	60	3	22.46	1594	191	8	2.69	0.11	2.80
cold	60	5	13.55	2443	268	9	1.49	0.05	1.54
cold	70	1	15.25	2091	123	35	0.90	0.26	1.15
cold	70	3	21.65	4479	441	46	2.13	0.22	2.35
cold	70	5	15.49	2705	209	27	1.20	0.15	1.35
cold	80	1	12.77	2030	138	33	0.87	0.21	1.08
cold	80	3	18.08	3088	297	21	1.74	0.12	1.86
cold	80	5	13.63	2234	160	36	0.98	0.22	1.20
cold	90	1	11.93	3678	354	39	1.15	0.13	1.27
cold	90	3	16.24	4421	392	40	1.44	0.15	1.59
cold	90	5	12.62	2471	262	18	1.34	0.09	1.43
cold	100	1	12.14	2935	219	36	0.91	0.15	1.05
cold	100	3	18.16	4414	379	32	1.56	0.13	1.69
cold	100	5	13.87	3042	323	26	1.47	0.12	1.59
hot	10	1	3.89	504	38	5	0.29	0.04	0.33
hot	10	3	5.32	511	42	4	0.44	0.04	0.48
hot	10	5	4.38	768	58	6	0.33	0.03	0.36
hot	20	1	15.73	4326	338	52	1.23	0.19	1.42
hot	20	3	13.68	3645	233	96	0.87	0.36	1.23
hot	20	5	15.59	4340	302	109	1.08	0.39	1.48
hot	30	1	15.94	2848	262	39	1.47	0.22	1.69
hot	30	3	13.08	2110	193	49	1.20	0.30	1.50
hot	30	5	16.20	1643	191	34	1.88	0.34	2.22
hot	40	1	15.69	3270	242	43	1.16	0.21	1.37
hot	40	3	13.09	3691	291	153	1.03	0.54	1.57
hot	40	5	16.24	2630	209	88	1.29	0.54	1.83
hot	50	1	15.74	1574	127	25	1.27	0.25	1.52
hot	50	3	13.32	1321	91	52	0.92	0.52	1.44
hot	50	5	15.04	1744	158	59	1.36	0.51	1.87
hot	60	1	17.35	3052	182	59	1.03	0.34	1.37
hot	60	3	14.04	1541	140	53	1.28	0.48	1.76
hot	60	5	15.64	1724	163	71	1.48	0.64	2.12

Table 11: Fraction of anchor reads that could not be aligned to the reference genome (in %). cond condition, g generation

		replicate		
cond	g	1	3	5
base	0	6.0	11.6	9.4
cold	10	8.9	11.7	11.1
cold	20	10.9	7.4	8.8
cold	30	11.9	10.4	10.1
cold	40	6.6	8.4	6.2
cold	50	6.3	5.8	7.6
cold	60	9.1	7.2	8.6
cold	70	5.3	6.8	7.0
cold	80	5.7	5.3	7.1
cold	90	6.9	7.2	6.6
cold	100	7.9	8.5	10.5
hot	10	8.9	9.2	12.1
hot	20	11.3	12.0	9.9
hot	30	17.1	17.3	12.1
hot	40	9.5	10.2	10.8
hot	50	7.4	7.5	8.2
hot	60	12.8	11.5	13.1

References

Robert Kofler, Kirsten-Andre Senti, Viola Nolte, Ray Tobler, and Christian Schlötterer. Molecular dissection of a natural transposable element invasion. *Genome Research*, 28(2):824–835, 2018.

D M Black, M S Jackson, M G Kidwell, and G A Dover. KP elements repress P-induced hybrid dysgenesis in *Drosophila melanogaster*. *The EMBO journal*, 6(13):4125–35, 1987.

Korise E Rasmusson, John D Raymond, and Michael J Simmons. Repression of hybrid dysgenesis in *Drosophila melanogaster* by individual naturally occurring P elements. *Genetics*, 133(3):605–622, 1993.

Sharmistha Majumdar and D C Rio. P transposable elements in *Drosophila melanogaster*. *Microbiol Spectrum*, pages 484–518, 2015.

Robert Kofler, Daniel Gomez-Sanchez, and Christian Schlötterer. PoPoolationTE2: Comparative Population Genomics of Transposable Elements Using Pool-Seq. *MBE*, 33(10):2759–2764, 2016.

L Weilguny and R Kofler. DeviaTE: Assembly-free analysis and visualization of mobile genetic element composition. *Molecular ecology resources*, 19(5):1346–1354, 2019.

Filip Wierzbicki, Florian Schwarz, Odontsetseg Cannalonga, and Robert Kofler. Novel quality metrics allow identifying and generating high-quality assemblies of pirna clusters. *Molecular Ecology Resources*, 2021a.

Filip Wierzbicki, Robert Kofler, and Sarah Signor. Evolutionary dynamics of piRNA clusters in Drosophila. *Molecular Ecology*, (August):1–17, 2021b.

Daniel Gebert, Lena K Neubert, Catrin Lloyd, Jinghua Gui, Ruth Lehmann, and Felipe Karam Teixeira. Large Drosophila germline piRNA clusters are evolutionarily labile and dispensable for transposon regulation. *Molecular Cell*, pages 1–14, 2021.
